# Rapid and zero-cost DNA extraction from soft-bodied insects for routine PCR-based applications

**DOI:** 10.1371/journal.pone.0271312

**Published:** 2022-07-15

**Authors:** Sumit Jangra, Amalendu Ghosh

**Affiliations:** Insect Vector Laboratory, Advanced Centre for Plant Virology, ICAR-Indian Agricultural Research Institute, New Delhi, India; National Bureau of Plant Genetic Resources, INDIA

## Abstract

Nucleic acid extraction is the first and foremost step in molecular biology studies. Extraction of DNA from small, soft-bodied insects is often time-consuming and costly. A fast, easy, and cost-effective DNA extraction method with greater yield and purity of DNA would aid in the rapid diagnostics, screening of large populations, and other routine PCR-based applications. The present study evaluated and standardized a rapid and zero-cost DNA extraction from soft-bodied small insects for routine molecular studies. Five rapid DNA extraction methods viz. extraction in sterile distilled water (SDW), 1X phosphate-buffered saline (PBS, pH 7.4), 1.4 M sodium chloride (NaCl), 20 mM ethylenediaminetetraacetic acid (EDTA, pH 8.0), and elution from blotted nitrocellulose membrane (NCM) were compared with standard CTAB extraction buffer and DNeasy^®^ Blood and Tissue Kit methods. The average yield, purity, storage stability, time, and cost of extraction were assessed for all the methods and compared. A method of DNA extraction by simply crushing the soft-bodied insects in SDW was ideal in terms of yield, purity, storability, and performing routine PCR-based applications including detection of pathogens from vector species. The extraction could be accomplished in 2.5 min only with zero-reagent cost. The method would be useful in rapid molecular diagnostics and screening large populations of soft-bodied insects.

## Introduction

The use of molecular biology tools in insect identification, invasion studies, and evolutionary genetics has increased over time [1–5]. The tools are indispensable not to only understanding gene functions but also in formulating novel management options. Molecular biology studies enable decoding the encoded information contained and conveyed via nucleic acid. Extracting the deoxyribonucleic acid (DNA) from insects is the fundamental first step in all these assays. The first protocol developed to extract nucleic acid from an insect was using cesium chloride [6]. Since then, several methods and kits have been standardized and/or commercially made available for isolation of insect DNA [7–11]. The central focus of all these methods was the high recovery of DNA, efficient removal of impurities and inhibitors, and high-throughput processing. Some of the most common methods include organic extraction, Chelex extraction, and solid-phase extraction. In the Chelex extraction method, the cellular components get stuck in the Chelex beads and DNA is available in the supernatant. The solid-phase extraction method utilizes spin-columns containing silica. DNA gets bound to silica in the column and other cellular components, and chaotropic salts are washed out [12]. Organic extraction involves addition and incubation in multiple different reagent solutions. This includes steps like lysis, phenol-chloroform separation, and ethanol precipitation. This method is most preferred in laboratories because it is cheap and yields higher quantities of DNA [13]. While selecting a DNA extraction method, multiple factors are to be considered including nature of the specimen, time, cost, yield, and quality of extracted DNA. PCR sensitivity is often considered to compare the applicability of different methods [14]. However, all these methods involved several steps for the separation and purification of DNA and are time-consuming. The commercial kits available for DNA extraction from small insects are costly too. A rapid, cost-effective method for DNA isolation from small, soft-bodied insects would help in faster diagnosis, screening of large populations, and other routine applications.

Aphids, thrips, and whitefly are tiny, soft-bodied insects, considered important sucking pests of crops. Many of them are vectors of plant diseases and pose quarantine threats due to their invasive nature and enormous economic damage potential. Around 4000 species of aphids have been reported and almost 50% of the insect-transmitted plant viruses are vectored by aphids [15]. The collective economic losses due to crop failure and measures to control aphids are around US$200 million annually [16]. Thrips damage crop plants by sucking plant sap and transmitting several genera of plant viruses [17]. Western flower thrips, *Frankliniella occidentalis* is reported to cause annual economic losses of around US$50 million in the Netherlands [18]. Tomato spotted wilt virus (TSWV) transmitted by *F*. *occidentalis* alone is reported to cause economic losses of around US$1 billion globally [19]. Yield losses of around 20–100% are known due to whitefly-transmitted begomoviruses [20–22]. Many of these aphids, thrips, and whitefly species are considered invasive and strict embargoes are being followed in international trade. Rapid diagnosis of these pests is essential for the decision support system and implementing the right interventions. Fast and simple DNA extraction from small individuals of aphids, thrips, and whitefly is important for rapid diagnosis. Smaller size, presence of cuticle, plant phenolic, and tannins impede the yield and purity of total DNA from small sap-sucking insects. Moreover, extraction of DNA from single aphids, thrips, or whitefly through conventional methods and kits is time, cost, and labour-intensive. The present study reports a rapid, simple, and zero-cost method for DNA extraction from small, soft-bodied insects. The method yields a higher quantity of DNA that is comparable to standard protocols and suitable for routine PCR applications.

## Materials and methods

### Insect specimen

Three small, soft-bodied insects viz. aphids (*Aphis gossypii* Glover, Hemiptera: Aphididae), whitefly (*Bemisia tabaci* Gennadius, Hemiptera: Aleyrodidae), and thrips (*Scirtothrips dorsalis* Hood, Thripidae: Thysanoptera) were considered for the study. Adults of *A*. *gossypii* and *S*. *dorsalis* were collected from cotton and chilli plants from the experimental fields of Indian Agricultural Research Institute (IARI), New Delhi, and carried to the laboratory. Adults of *B*. *tabaci* were collected from a stock culture maintained at Advanced Centre for Plant Virology, IARI, New Delhi. Insects were visualized under a stereomicroscope and the size of the specimens was measured. Individuals of uniform size and life-stage (adult female) of respective insect species were considered for DNA extraction by different methods.

### DNA extraction from a single insect

DNA was extracted from single insects by seven different methods. Methods were broadly categorized as standard and rapid methods. The standard methods included DNA extraction using cetyltrimethylammonium bromide (CTAB) extraction buffer [23] and DNeasy^®^ Blood and Tissue Kit (Qiagen, Germany). Rapid DNA extraction from individual specimens was carried out using five methods viz. extraction in sterile distilled water (SDW), 1X phosphate-buffered saline (PBS, pH 7.4), 1.4 M sodium chloride (NaCl), 20 mM ethylenediaminetetraacetic acid (EDTA, pH 8.0), and elution from blotted nitrocellulose membrane (NCM).

### DNA extraction using standard methods

#### CTAB extraction buffer

DNA was extracted from individual specimens in CTAB extraction buffer. Briefly, 10 ml CTAB extraction buffer contained 3.5 ml of 10% CTAB, 2.8 ml of 5 M NaCl, 1 ml of 1 M Tris-HCl, 400 μl of 0.5 M EDTA (pH 8.0), 20 μl β-mercaptoethanol, and 2.28 ml SDW. Single adult females of respective species were taken in l.5 ml micro-centrifuge tubes separately and crushed in 100 μl of extraction buffer using micro-pestles. The lysate was incubated at 65°C for 1 hr with intermittent vortexing at every 10 min interval. An equal volume of chloroform: isoamyl alcohol (24: 1) was added to the lysate, mixed, and centrifuged at 14,000 x*g* for 15 min. The upper aqueous layer was transferred to a new micro-centrifuge tube. A 0.8 volume of chilled isopropanol was added and kept at -20°C for 30 min. The DNA was pelleted by centrifuging at 14,000 x*g* for 10 min. The supernatant was decanted gently without disturbing the pellet. The DNA pellet was washed with 70% ethanol. The residual ethanol was removed by allowing the samples to dry at room temperature. The pellet, if any was dissolved in 20 μl SDW and used for yield, purity assessment, and PCR amplification.

### DNeasy^®^ Blood and Tissue Kit

Total DNA was extracted from single adult female individuals using DNeasy^®^ Blood and Tissue Kit following the manufacturer’s protocol. Briefly, a single specimen was taken in a 1.5 ml microcentrifuge tube and crushed in 180 μl of ATL buffer using a sterile micropestle. 20 μl of Proteinase K was added and the lysate was incubated at 56°C for 1 hr. 200 μl of AL buffer and 200 μl molecular grade ethanol were added and mixed thoroughly by vortexing. The mixture was transferred to a DNeasy^®^ Mini spin column and centrifuged at 6000 x*g* for 1 min. The flow-through was discarded and centrifuged at 6000 x*g* for 1 min after the addition of 500 μl buffer AW1. The flow-through was discarded and 500 μl of buffer AW2 was added. The column was centrifuged at 20,000 x*g* for 3 min, placed in a new fresh 1.5 ml micro-centrifuge tube, and incubated at room temperature for 10 min. The DNA was eluted by adding 20 μl of Buffer AE at the center of the column followed by centrifugation at 6000 x*g* for 1 min. The eluted DNA was used for yield, purity assessment, and PCR amplification.

### Rapid DNA extraction

For simple and rapid DNA extraction, individual insects were collected in 1.5 ml microcentrifuge tubes and crushed separately in 20 μl of SDW, 1X PBS (containing 0.137 M NaCl, 0.0027 M KCl, 0.01 M Na_2_HPO_4_, and 0.0018 M KH_2_PO_4_), 1.4 M NaCl, and 20 mM EDTA using sterile micro-pestles. All the lysates were incubated at 98°C for 2 min in a water bath and assessed for purity, quantity, and PCR applications. DNA extraction was performed in 10 replicates.

In the case of NCM, individual adults were blotted on the membrane using sterile micro-pestles. The blotted membrane was air-dried and stored at room temperature. A single dot was cut and taken in a microcentrifuge tube. DNA was eluted in 20 μl SDW by incubating at 98°C for 2 min and assessed for purity, quantity, and PCR applications.

### Assessment of quantity, purity, and storability

The quantity and purity of the DNA extracted through different methods as described above were assessed in a spectrophotometer (NanoDrop^TM^ One, Thermo Fisher Scientific). To assess the storage stability, the DNA extracted through different methods was stored at 4°C except for NCM. Purity and quantity were assessed at weekly intervals. The NCM was stored at room temperature, eluted at weekly intervals, and assessed for purity and quantity. Concentration (ng/μl), absorbance ratio at 260/280 (A_260/280_), and 260/230 (A_260/230_) of extracted DNA were recorded in three technical replicates each time in a spectrophotometer and their average was considered.

### Fluorometric quantification of DNA

The concentration of DNA was further quantified using QuantiFluor^®^ ONE dsDNA System (Promega, USA). 2 μl of the template was added to 200 μl of QuantiFluor^®^ ONE dsDNA dye in a 0.5 ml microcentrifuge tube in three replicates. The samples were mixed well and incubated at room temperature for 5 min. The Qubit™ 3 Fluorometer (Thermo Fisher Scientific) was calibrated using the blank and standard. The fluorescence bound to dsDNA was recorded and the concentration of DNA was measured in ng/μl.

### PCR amplification of COI loci

PCR was carried out with template DNA isolated through different methods to compare the sensitivity of the extracted DNA. The universal primer pair LCO 1490 and HCO 2198 [24] for amplification of mitochondrial cytochrome oxidase subunit I (COI) was used in the assay. A 25 μl PCR mixture contained 1X DreamTaq buffer (Thermo Fisher Scientific), 0.4 μM each forward and reverse primer (GCC Biotech, India), 260 μM dNTP mix (Thermo Fisher Scientific), ~20 ng DNA template, and 2 units of DreamTaq DNA polymerase (Thermo Fisher Scientific). PCR was performed in a Prima-Duo^TM^ thermal cycler (Himedia, India) with initial denaturation at 94°C for 5 min followed by 35 cycles of denaturation at 94°C for 30 sec, annealing at 45°C for 1 min 30 sec, extension at 72°C for 1 min, and a final extension at 72°C for 10 min. The amplified PCR products were resolved on 1% agarose gel stained with GoodView (BR Biochem, India) and visualized in a gel documentation system (MaestroGen Inc, Taiwan) with a 100 bp Plus DNA ladder (Thermo Fisher Scientific).

To assess the sensitivity of stored DNA extracted through different methods, PCR was performed at weekly intervals with DNA templates stored at 4°C or eluted from NCM. The DNA templates that did not show amplification in PCR were not tested in the following weeks. Representative amplicons were sequenced to substantiate the quality.

### Pathogen detection using DNA extracted through SDW

Detection of the pathogen was attempted from the template DNA extracted from *B*. *tabaci* using SDW, PBS, kit and CTAB methods. Based on the results of PCR amplification of COI loci, the other methods were not included for pathogen detection. *B*. *tabaci* was exposed to chilli leaf curl virus (ChiLCV, *Begomovirus*)-infected chilli plants for 24 hrs. DNA was extracted from a single and a group of five adults of *B*. *tabaci*. PCR was performed using ChiLCV-specific primers, AG149F-AG150R [25]. The 25 μl PCR reaction comprised 1 μl of template DNA (~ 20 ng), 0.4 μM of each forward and reverse primers (GCC Biotech), 260 μM dNTP mix (Thermo Fisher Scientific), and 2 units of DreamTaq DNA polymerase (Thermo Fisher Scientific). PCR was performed in a Prima-Duo thermal cycler with an initial denaturation at 95°C for 5 min followed by 35 cycles of denaturation at 95°C for 30 sec, annealing at 53°C for 1 min 30 sec, extension at 72°C for 40 sec, and a final extension at 72°C for 10 min. The amplified PCR products were resolved on 2% agarose gel stained with GoodView and visualized in a gel documentation system with a 1 kb Plus DNA ladder (Thermo Fisher Scientific).

### Statistical analysis

The mean DNA quantity obtained from the spectrophotometer and fluorometer readings for different methods was analyzed by Duncan’s multiple mean comparison test using XLSTAT 2014.5.03 software. The mean difference was considered statistically significant at a 95% confidence level (p<0.05).

### Time and cost involvement

The time required to complete DNA extraction from a single individual of the three insect species was calculated as the time required to complete the different steps in the extraction procedure including the sample crushing time. The time required to prepare the reagents and buffers was not included.

The cost of DNA extraction for each method was estimated based on the cost of the DNA extraction kit and chemical reagents (Thermo Fisher Scientific, USA). The NCM was cut into pieces of 0.5 x 0.5 cm and the price of the unit area was calculated. The cost of the disposable items like centrifuge tubes, tips, and equipment such as micropipettes, centrifuge, vortex, water bath, refrigerator, etc. required in different extraction methods was excluded.

## Results

### Assessment of quantity and purity of extracted DNA

The size of the three insect specimens viz. aphids, whitefly, and thrips was about 1.2 mm, 1 mm, and 0.9 mm in length, respectively. Individuals of the same size and life stage for each insect species were considered for DNA extraction. The apparent DNA yield and purity that were measured in NanoDrop^TM^ One varied significantly with the methods and insect species.

**Fig 1:**
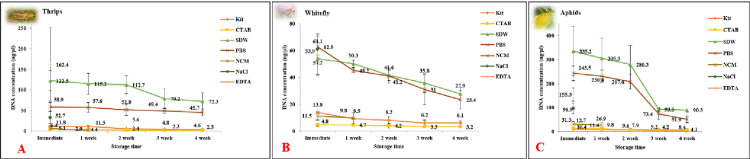
Average DNA concentration extracted through different methods over a period of four weeks. DNA was extracted from individuals **A**) aphids, **B**) thrips, and **C**) whitefly. A gradual decline in average DNA concentration was observed in all the methods. In case of aphids and whitefly, the decline was faster in SDW and PBS. However, it was slower than the standard methods in thrips. The error bars are standard error of means (SEm), n = 15. In case of aphids the decline in DNA concentration was statistically significant in kit (p-value 0.005; F valve 7.24; df 14), SDW (p-value 0.05; F value 2.93; df 14), and PBS (p-value 0.001; F value 12.83; df 14). In whitefly, statistically significant decline was observed in kit (p valve 0.05; F value 3.33; df 14), SDW (p-value 0.02; F value 4.69; df 14), and PBS (p-value 0.02; F value 4.53; df 14). While in case of thrips statistically significant decline was observed only in the kit (p-value 0.01; F value 5.13; df 14).

In aphids, the highest DNA concentration of 335.2 (± 102.8) ng/μl was observed in SDW (Fig 1A). A mean A_260/280_ ratio of 1.8 (± 0.05) indicated the good quality of the extracted DNA with lower contaminations of RNA and protein (Table 1). However, a low A_263/230_ (0.9 ± 0.03) indicated carbohydrate contamination. The next best yield was obtained in PBS (243.5 ± 30.4 ng/μl) followed by EDTA (155.3 ± 27.5), and NaCl (96.9 ± 20.6). Although the purity of DNA extracted in PBS was good based on A260/280 (1.8 ± 0.01), it was inferior in EDTA and NaCl. A very low A_260/230_ of DNA extracted in EDTA and NaCl indicated high contaminations of EDTA and salts. The purity of DNA extracted by blotting in NCM and elution was comparable to standard methods. However, the quantity of DNA eluted from NCM was as little as 31.3 (± 10.4) ng/μl. The quantity of DNA extracted through standard methods was almost 30-fold lower than the SDW method. The mean concentration of DNA in CTAB method and kit was 12.7 (± 4.1) and 10.4 (± 1.2) ng/μl, respectively. The mean A_260/280_ of DNA extracted in CTAB method (2.1 ± 0.01) and kit (2.0 ± 0.01) indicated the presence of a higher amount of RNA than in the rapid methods. The mean A_260/230_ for CTAB and kit was equivalent to SDW and PBS methods.

**Table 1:** Assessment of quantity and quality of DNA up to four weeks.

**Aphids**	**Immediate**			**One week**			**Two weeks**			**Three weeks**			**Four weeks**		
**Method**	A_260/280_	A_260/230_	PCR	A_260/280_	A_260/230_	PCR	A_260/280_	A_260/230_	PCR	A_260/280_	A_260/230_	PCR	A_260/280_	A_260/230_	PCR
**Kit**	2.0 ±0.01	1.5 ±0.03	+ +	2.1±0.2	1.2 ±0.1	+ +	3.3 ±0.9	0.1 ±0.04	+ +	4.1 ±0.8	0.1 ±0.03	+ +	3.0 ±0.2	0.1 ±0.01	+
**CTAB**	2.1 ±0.01	1.0 ±0.3	+ +	2.8 ±0.4	1.1 ±0.3	+ +	3.5 ±1.2	2.3 ±1.7	+ +	2.5 ±0.1	0.2 ±0.1	+ +	2.1 ±0.1	0.6 ±0.3	+
**SDW**	1.8 ±0.05	0.9 ±0.03	+ +	1.7 ± 0.1	1.1 ±0.1	-	1.4 ±0.1	0.5 ±0.03		1.5 ±0.1	0.5 ±0.04		1.5 ±0.1	0.4 ±0.04	
**NCM**	1.7 ±0.1	0.7 ±0.01	+	1.8 ±0.1	0.7 ±0.1	-	Not considered for further studies								
**PBS**	1.8 ±0.01	1.0 ±0.04	+ +	1.9 ±0.03	1.4 ±0.8	++	1.5 ±0.03	0.5 ±0.03	+	1.5 ±0.1	0.6 ±0.04	+	1.5 ±0.03	0.5 ±0.03	+
**EDTA**	1.6 ±0.1	18.2 ±13.7	-	Not considered in following weeks											
**NaCl**	1.6 ±0.1	0.6 ±0.04	-												
**Whitefly**	**Immediate**			**One week**			**Two week**			**Three week**			**Four week**		
**Method**	A_260/280_	A_260/230_	PCR	A_260/280_	A_260/230_	PCR	A_260/280_	A_260/230_	PCR	A_260/280_	A_260/230_	PCR	A_260/280_	A_260/230_	PCR
**Kit**	3.2 ±0.2	0.1 ±0.01	+ +	2.8 ±0.1	0.3 ±0.1	+ +	2.6 ±0.2	0.2 ±0.03	+ +	2.2 ±0.3	0.1 ±0.02	+ +	1.5 ±0.1	0.3 ±0.04	+ +
**CTAB**	3.3 ±0.5	0.9 ±0.1	+ +	2.5 ±0.1	1.25 ±0.3	+ +	2.1 ±0.3	0.3 ±0.1	+ +	2.1 ±0.3	0.3 ±0.1	+ +	2.3 ±0.4	0.3 ±0.1	+ +
**SDW**	1.5 ±0.1	0.7 ±0.1	+ +	1.6 ±0.2	0.8 ±0.1	+	1.5 ±0.05	0.4 ±0.02	+	1.4 ±0.03	0.4 ±0.01	-	1.5 ±0.04	0.5 ±0.02	
**NCM**	1.1 ±0.2	0.3 ±0.1	+ +	1.3 ±0.2	0.3 ±0.1	-	Not considered for further studies								
**PBS**	1.2 ±0.1	0.7 ±0.1	+ +	1.3 ±0.1	0.7 ±0.1	+ +	1.6 ±0.1	1.6 ±0.5	+ +	1.6 ±0.02	1.5 ±0.3	+ +	1.5 ±0.04	1.3 ±0.2	+ +
**EDTA**	1.3 ±0.2	3.0 ±8.7	-	Not considered in following weeks											
**NaCl**	1.4 ±0.2	0.2 ±0.1	-												
**Thrips**	**Immediate**			**One week**			**Two week**			**Three week**			**Four week**		
**Method**	A_260/280_	A_260/230_	PCR	A_260/280_	A_260/230_	PCR	A_260/280_	A_260/230_	PCR	A_260/280_	A_260/230_	PCR	A_260/280_	A_260/230_	PCR
**Kit**	4.6 ±0.6	0.1 ±0.01	+ +	3.6 ±1.4	0.1 ±0.01	+ +	2.8 ±0.7	0.1 ±0.01	+ +	2.1 ±0.1	0.2 ±0.02	+ +	2.2 ±0.2	0.4 ±0.2	+ +
**CTAB**	5.5 ±1.5	0.6 ±0.1	+ +	3.5 ±0.7	0.1 ±0.01	+ +	3.8 ±1.0	0.2 ±0.04	+ +	1.6 ±0.1	0.4 ±0.1	+ +	1.6 ±0.2	0.4 ±0.1	+ +
**SDW**	1.6 ±0.1	0.4 ±0.1	+ +	1.4 ±0.4	0.7 ±0.5	+ +	1.6 ±0.1	0.2 ±0.01	+ +	0.8 ±0.1	0.2 ±0.01	+ +	1.5 ±0.04	0.2 ±0.03	+ +
**NCM**	0.4 ±0.1	0.2 ±0.03	+ +	1.8 ±0.1	0.3 ±0.01	-	Not considered for further studies								
**PBS**	0.8 ±0.2	0.4 ±0.1	+ +	1.0 ±0.1	0.2 ±0.03	+ +	1.3 ±0.1	0.3 ±0.1	+ +	1.3 ±0.1	0.3 ±0.1	+ +	1.3 ±0.1	0.4 ±0.04	+ +
**EDTA**	1.1 ±0.1	0.3 ±0.1	-	Not considered in following weeks											
**NaCl**	1.1 ±0.1	0.5 ±0.1	-												

+ +: sharp amplicon; +: faint amplification;−: no amplification.

In thrips, although the highest concentration (162.4 ± 89.1 ng/μl) of DNA was recorded with EDTA, mean A_260/280_ (1.1 ± 0.1) and A_260/230_ (0.3 ± 0.1) indicated contaminations of protein and EDTA (Fig 1B and Table 1). A moderate yield of DNA was recorded in NaCl but the purity was inferior due to possible contaminations of salts and proteins. The quantity of DNA extracted from single thrips in CTAB method, kit, and NCM was very low ranging from 5.1–11.8 ng/μl. A very high A_260/280_ in the kit (4.6 ± 0.6) and CTAB (5.5 ± 1.5) indicated RNA contaminations. Whereas, the DNA concentration in SDW (122.5 ±24.2 ng/μl) was around 11-fold and 25-fold higher than the commercial kit and CTAB. An A_260/280_ of 1.6 (±0.1) also indicated better purity. The next best DNA concentration was recorded in PBS (58.9 ±10.2 ng/μl).

In whitefly, a similar trend was observed like aphids and thrips. The DNA concentration was higher in PBS (62.9 ± 7.9 ng/μl) and SDW (53.9 ± 2.5) (Fig 1C). However, the mean A_260/280_ and A_260/230_ indicated protein and carbohydrate contaminations (Table 1). The concentration of extracted DNA was 5-12-fold lower in the kit and CTAB method. A higher A_260/280_ and lower A_260/230_ indicated contaminations of RNA, phenols, and carbohydrates in CTAB method and kit. Although the concentration of DNA eluted from NCM was as low as 11.5 (± 3.2) ng/μl, the purity was comparable with PBS and SDW methods. The mean DNA concentration in EDTA and NaCl (57.2 ± 15.4 and 61.1 ± 11.1 ng/μl) was almost equivalent to PBS and SDW. However, the mean A_260/280_ and A_260/230_ indicated contaminations of EDTA and salts in extracted DNA. In general, A_260/230_ of the extracted DNA was low irrespective of the methods that indicated the presence of a higher amount of carbohydrates and phenols interfering with the purity of extracted DNA in these insect species.

**Fig 2:**
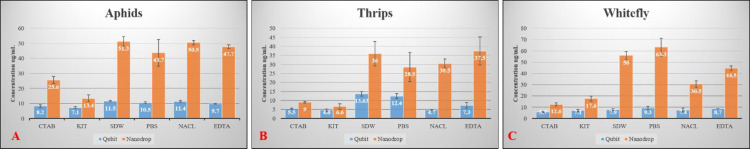
Comparison of two different DNA quantification methods. The DNA concentration of **A)** aphids, **B)** thrips, and **C)** whitefly extracted through different methods was measured using Qubit™ 3 Fluorometer and NanoDropTM One. The error bars are standard errors of means.

As the quantification of DNA based on UV absorbance-based values was apparent, it was further corroborated by a fluorescence-based measurement to provide more accuracy and reliability to the data. QuantiFluor^®^ ONE dsDNA System was used to quantify the dsDNA in Qubit™ 3 Fluorometer. The fluorescence-based quantification of DNA was comparatively lower than the DNA concentration measured in NanoDrop^TM^ One (Fig 2). Although the apparent DNA yield in instant preparations was overestimated in NanoDrop^TM^, the DNA concentration remained significantly higher for SDW and PBS than the CTAB and kit methods in Qubit™ 3 Fluorometer.

If summarized, SDW and PBS yielded better quantity of DNA than the commercial kit and CTAB method in all three insect specimens. DNA extracted in EDTA and NaCl contained impurities. The purity of DNA eluted from NCM was comparable but the concentration was too low.

### PCR sensitivity of extracted DNA

PCR sensitivity was considered to compare the applicability of the template DNA extracted through different methods. The PCR sensitivity assay was consistent with the purity of DNA measured in the spectrophotometer. In aphids, PCR amplification immediately after DNA extraction from a single insect yielded sharp amplicons of 750 bp using template DNA extracted through SDW, PBS, CTAB, and kit (S1 Fig). While a faint amplicon was observed with DNA extracted through NCM. The purity of extracted DNA in EDTA and NaCl was inferior based on A_260/280_, and A_260/230_. In consistent with that, no amplification was observed from DNA extracted using NaCl and EDTA.

In the case of thrips, PCR sensitivity of the template DNA extracted from a single insect through CTAB, kit, PBS, SDW, and NCM was equivalent (S2 Fig). PCR amplification immediately after extraction showed sharp amplicons of 750 bp on an agarose gel. However, no amplification was observed in PCR using template DNA extracted in NaCl and EDTA. A similar trend was recorded in the case of whitefly (S3 Fig). Unlike in aphids, a comparable amplicon was visualized on agarose gel post PCR with a DNA template eluted from blotted NCM. No amplification was observed from whitefly DNA extracted using NaCl and EDTA.

Altogether, the assay showed comparable PCR sensitivity of the extracted DNA using SDW, PBS, CTAB, and kit methods for all three insect specimens. DNA extracted in EDTA and NaCl could not be amplified in PCR, hence not suitable for routine applications. Although DNA eluted from blotted NCM yielded desired PCR amplification for whitefly and thrips DNA, it was less sensitive for aphids DNA in comparison to other methods. The obtained PCR amplicons were further sequenced by Sanger sequencing to verify that the intended marker was amplified. The sequencing results indicated that the method will be suitable for genetic studies and characterization. The GenBank accession numbers of the sequences have been provided under the data availability statement.

### Storage stability of the extracted DNA

The DNA templates from individual specimens of all the three insect species were extracted through different methods and assessed for their storage stability. DNA templates extracted in NaCl and EDTA did not show any PCR amplification immediately after extraction for any of the insect specimens. Hence, these templates were not considered for further assessments for storage stability. There was a gradual decline in quantity and purity with time for the rest of the DNA templates.

In aphids, a 3.7- and 4.6-fold decline in DNA concentration was recorded in SDW (243 to 51.9 ng/μl) and PBS over four weeks. However, a slower decline of 2.5-fold (10.4 to 4.1 ng/μl) and 1.5-fold in DNA concentration was observed in the case of kit and CTAB methods, respectively. Unlike other insect specimens, the template in SDW did not yield any amplification in PCR post one week. The aphids contained more amount of plant sap than the other two insect species and the template isolated in SDW was pigmented. The presence of pigments and phenols of the plant sap in DNA template probably inhibited the PCR sensitivity. PCR sensitivity of the DNA extracted in PBS showed a gradual decrease second week onwards (Fig 1A). The presence of nuclease and reactive oxygen species (ROS) are probably responsible for decreased PCR sensitivity over time. The template eluted from NCM produced very faint amplification in PCR immediately after extraction and no amplification was observed post one week. This might be due to the degradation of DNA at room temperature. There was no significant change in PCR sensitivity for the DNA extracted in the kit and CTAB methods for up to four weeks.

A similar trend was observed in the case of thrips. There was a gradual decline in the quantity and purity of template DNA extracted through different methods over time. Unlike in aphids and whitefly, there was no decline in PCR sensitivity of DNA extracted in SDW. DNA templates extracted through CTAB, kit, PBS, and SDW showed comparable PCR sensitivity and produced sharp amplicons of 750 bp for up to four weeks (Fig 1B). In the case of kit and CTAB, the mean DNA concentration dropped by 2.6-fold (11.8 to 4.4 ng/μl) and 2.2-fold (5.5 to 2.4 ng/μl), respectively over four weeks. Whereas, the decline of DNA concentration was slower in the rapid methods. Over four weeks, there was a 1.6- and 1.2-fold decrease in DNA concentration, respectively for SDW and PBS methods. However, no PCR amplification was observed with the DNA template eluted from NCM post one week.

In the case of whitefly, there was a gradual decrease in the quantity and purity of DNA templates in all the methods. A 1.5–2.3-fold decline in mean quantity was observed in the case of standard methods post four weeks of extraction. Whereas, a 1.9-fold (53.9 to 27.9 ng/μl) decline in DNA concentration in SDW and a 2.6-fold (62.9 to 23.4 ng/μl) in PBS were recorded. There was no significant change in PCR sensitivity of the DNA templates in CTAB, kit, and PBS. PCR amplification showed sharp amplicons of 750 bp up to four weeks post-extraction (Fig 1C). PCR sensitivity of DNA template in SDW declined with time and no amplification was observed post-second week. DNA eluted from blotted NCM showed no amplification from one week onwards.

Altogether, the results indicated that the storage stability was better in CTAB, kit, and PBS. DNA template in SDW was suitable for routine PCR amplification for up to 1–2 weeks if stored at 4°C. However, the storage stability of the DNA template extracted in SDW from single thrips was equivalent to PBS, CTAB, and kit.

### Pathogen detection using DNA template extracted through rapid methods

**Fig 3:**
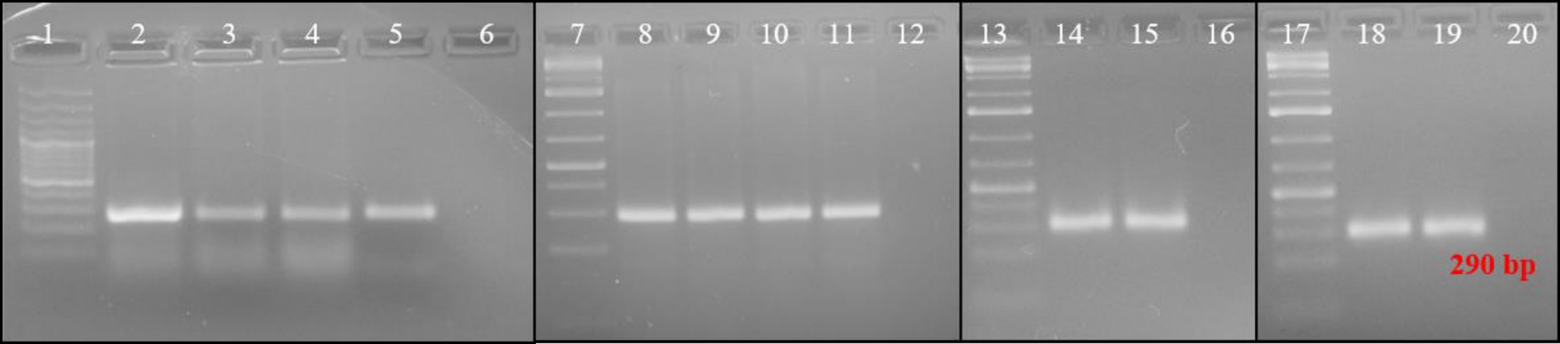
Detection of pathogen from template DNA extracted in SDW, PBS, kit, and CTAB methods. DNA was extracted from a group of five whiteflies and used for PCR assay. A sharp ChiLCV-specific amplicon was observed for DNA templates extracted in SDW and PBS. However, no amplification was observed for the DNA template from a single adult fly. Lanes 1, 7, 13, 17: 1 kb plus DNA ladder, lanes 2–5: amplification of ChiLCV from DNA extracted in SDW, lanes 8–11: amplification of ChiLCV from DNA extracted in PBS, lanes 14–15: amplification of ChiLCV from DNA extracted in kit, lanes 18–19: amplification of ChiLCV from DNA extracted in CTAB method, lanes 6, 12, 16, 20: non-template control.

PCR with ChiLCV-specific primers AG149F-AG150R using template DNA extracted in SDW and PBS produced amplicons of 290 bp (Fig 3) as efficient as templates in kit and CTAB methods. This signifies the utility of the methods. However, there was no ChiLCV-specific amplification in PCR with DNA template from single *B*. *tabaci*. This might be due to the low concentration of viral DNA in the template extracted from a single insect.

### Time and cost effectivity of DNA extraction methods

The time taken to extract DNA using standard CTAB extraction buffer and DNeasy^®^ Blood and Tissue kit was around 2 hrs 55 min and 1 hr 33 min, respectively. DNA could be extracted in SDW, PBS, EDTA, NaCl, and NCM within 2.5 min (Table 2). The time for preparation for buffer and arranging reagents was not included.

**Table 2:** Time and cost involvement for DNA extraction through different methods.

Sl. No.	Technique	Reagents and Chemicals	Time required	Reagent cost per reaction (in US$)
**1.**	**CTAB**	CTAB, 5 M NaCl, 0.5 M EDTA, 1 M Tris-HCl, and β-mercaptoethanol	2 hrs 55 min	0.15
**2.**	**Kit**	As provided with the kit	1 hr 33 min	3.35
**3.**	**SDW**	SDW	2.5 min	0.0
**4.**	**PBS**	PBS	2.5 min	0.0016
**5.**	**NCM**	NCM and SDW	2.5 min	0.012
**6.**	**NaCl**	1.4 M NaCl	2.5 min	0.011
**7.**	**EDTA**	0.02 M EDTA	2.5 min	0.0058

The cost of the extraction ranged from nil to US$3.35 (Table 2). The extraction of DNA using the kit and CTAB was around US$ 3.35 and 0.15, respectively. The extraction cost from PBS, EDTA, NaCl, and NCM were US$0.0016, 0.0058, 0.011, and 0.012, respectively. Only the reagent cost was included and the cost of disposable items was excluded. The cost for extraction of DNA in SDW was considered nil. The results indicated that extraction through kit was the most expensive and extraction using SDW was the cheapest (zero-cost).

## Discussion

To date, several protocols are known for the extraction of DNA from small insects [26–29]. Most of these methods take time for the separation of impurities and contaminations. Commercial kits are more expensive than conventional methods [30]. The yield and purity of DNA vary with the type of insect specimens and protocol [11, 31]. The current intention is to reduce the time and cost involved in DNA extraction without much compromise on the yield and quality. Extraction of high-quality DNA from a single tiny, soft-bodied insect is always challenging due to its small size, lower biomass, and the presence of excessive plant sap in the body. In this study, we reported a rapid and zero-cost extraction of DNA from small, soft-bodied, sap-sucking insects. Five rapid DNA extraction methods viz. extraction in SDW, PBS, NaCl, EDTA, and elution from blotted NCM were compared with the standard CTAB method and DNeasy^®^ Blood and Tissue Kit. Adults of aphids, thrips, and whitefly were chosen as test specimens as they are smaller in size, soft-bodied, and considered important crop pests. The yield, purity, PCR sensitivity, and storage stability of the DNA extracted from single individuals of aphids, whitefly, and thrips were assessed.

Among the different rapid and standard methods tested in the present study, DNA extraction in SDW and PBS produced a higher yield and purity of DNA than in CTAB extraction buffer and DNeasy^®^ Blood and Tissue Kit. The quantification of DNA was further checked using a dsDNA-binding fluorescent dye in a fluorometer. The UV-absorbing impurities in instant DNA preparations might be involved in an apparently higher yield of DNA. The low yield of DNA using the kit was due to the loss of DNA that was either not initially adsorbed or irreversibly bound on the column surface [32–34]. Loss of DNA in repeated washing and resuspension might also contribute to low DNA yield in standard methods. The insects taken in the present study were very small and initial biomass was low, in such cases, the losses in DNA due to residual CTAB in the final extracts are likely to be more severe [35, 36]. In the present study, a higher yield of DNA through rapid methods was attributed to no loss in the process of extraction. A similar rapid method for insect DNA isolation by heating in alkaline buffer yields a good amount of DNA in 20 min [31]. The genomic DNA extraction method from the mouse by heating in an alkaline lysis solution comprising sodium hydroxide (pH 12) and Tris-HCl (pH 5) for 10 min has been reported by Truett and colleagues [37]. In the present study, we undertook the lysis by heating at 98°C in SDW for 2 min. Cell lysis by heating works well for bacterial cells [38–40]. We have implemented the same approach for small, soft-bodied insects.

The purity of the DNA extracted from individual aphids, thrips, and whitefly in SDW and PBS was superior to the DNA extracted through the standard CTAB method and kit. The mean A_260/280_ of the DNA extracted through SDW indicated the purity over standard methods. PCR sensitivity of the extracted DNA was tested to check the suitability in routine molecular assays. The PCR amplification using DNA extracted in SDW and PBS was comparable to CTAB and kit. The viral DNA present in the insect could also be amplified from DNA templates extracted in SDW and PBS. This signifies the utility of the instant methods in rapid diagnostics as well as in routine molecular assays. PCR with template DNA eluted from NCM was less sensitive to PCR indicated by a thinner band. No amplification was observed in the case of EDTA and NaCl extraction. EDTA is a strong chelator of Mg^2+^ ions. Due to the presence of a high level of residual EDTA, Mg^2+^ becomes unavailable for *Taq* DNA Polymerase to carry out the reaction [41]. The Na^+^ ions are also strong inhibitors of Mg^2+^ and stabilize the DNA duplex making the unwinding difficult and hence preventing the amplification [42] that clarifies the non-suitability of DNA template in NaCl for PCR amplification.

The quantity of extracted DNA gradually decreased over time irrespective of the methods. The possible explanation for this gradual decline in yield and purity is the degradation of DNA over time [43, 44]. The reduction in concentration and purity was faster in the case of rapid approaches than standard methods. This may be due to the presence of nucleases and ROS in the lysate. The presence of alkaloids and phenolics affects the purity of the DNA and therefore hinders PCR amplification [34, 45]. However, in the case of thrips, sharp PCR amplification with DNA templates extracted in SDW even after four weeks of extraction indicated strong PCR sensitivity of the extracted DNA. DNA extraction in SDW might not provide longer storage stability but it would be advantageous for immediate applications, maybe up to 1–2 weeks. Besides PCR, we also recorded high sensitivity of the DNA extracted in SDW in recombinase polymerase amplification (RPA) [5] and polymerase spiral reaction assay [46] that use enzymes other than *Taq* DNA polymerase. Whereas, DNA extracted in PBS showed high PCR sensitivity comparable to standard methods even after four weeks of storage. PBS extraction could be an ideal alternative to SDW extraction where longer storage stability is required. The DNA extracted through NCM showed a sharp amplicon when PCR assay was done immediately after DNA extraction. Zou and colleagues [47] also reported rapid capture and retention of DNA from plants, animals, and microbes on untreated cellulose-based paper. However, in the present study, no PCR amplification was recorded with DNA eluted from NCM in the following weeks. The insect lysate and tissue blotted in the NCM might have degraded with time when stored at room temperature [48, 49].

The time required to crush the insect sample and lysis in SDW or PBS was around 2.5 min only. To our knowledge, this is the fastest protocol for extraction of DNA from a single individual of soft-bodied, small insects reported to date. Serval low-cost DNA extraction methods are known where the estimated cost ranges from US$0.1–0.5 [50, 51]. Whereas, the present study reports the zero-cost protocol for extraction of insect DNA.

## Conclusion

In conclusion, the extraction of DNA from a single soft-bodied, small insect by simple lysis in SDW is a rapid, easy, and zero-cost procedure. It yields a higher amount of DNA of acceptable quality and is stable in storage at 4°C for a short period. Extraction in PBS provides an equivalent quantity and purity of DNA with longer storage stability. The procedure does not require any sophisticated laboratory equipment and does not produce hazardous waste. The rapid, zero-cost method for DNA extraction from soft-bodied, small insects will be useful in rapid diagnosis, screening of large populations, and routine molecular assays.

## Supporting information

S1 Raw images (PDF)

S1 Fig (DOCX)

S2 Fig (DOCX)

S3 Fig (DOCX)
