# Supplementary material for: Rapid and zero-cost DNA extraction from soft-bodied insects for routine PCR-based applications
Source: PLoS One. 2022 Jul 15;17(7):e0271312. doi: 10.1371/journal.pone.0271312 (PMC9286237; doi:10.1371/journal.pone.0271312)
Supplement: S1 Raw images — (PDF) [file pone.0271312.s001.pdf]

Aphids

Gel images of PCR amplification immediately after DNA extraction in Aphids

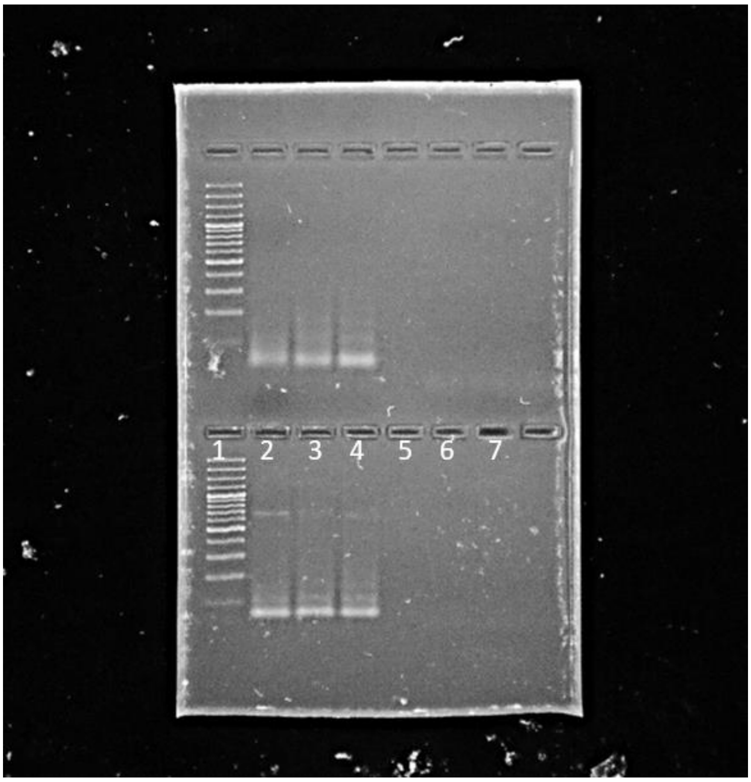

Lane 1: 100 bp plus DNA ladder; 2, 3, and 4: amplicon of DNA extracted through SDW; 5, 6, and 7: amplicon of DNA extracted through NaCl

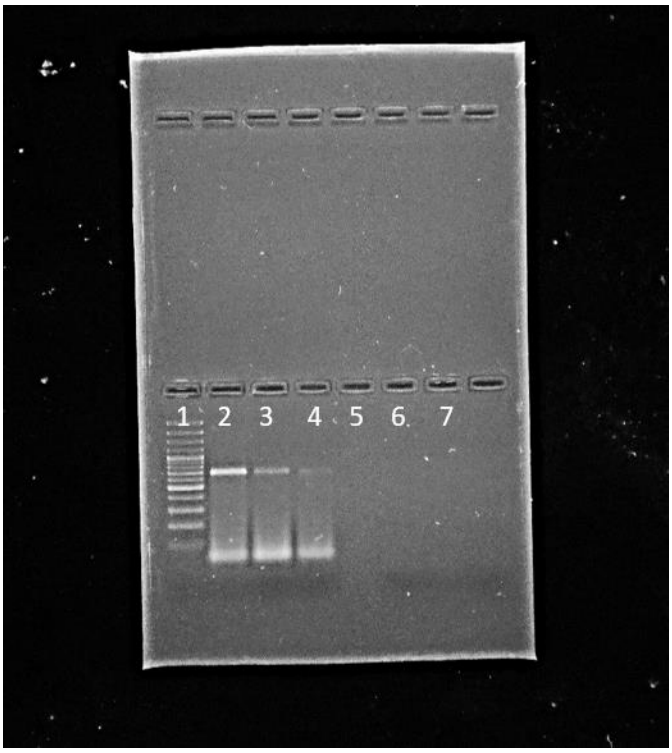

Lane 1: 100 bp plus DNA ladder; 2, 3, and 4: amplicon of DNA extracted through PBS; 5, 6, and 7: amplicon of DNA extracted through EDTA

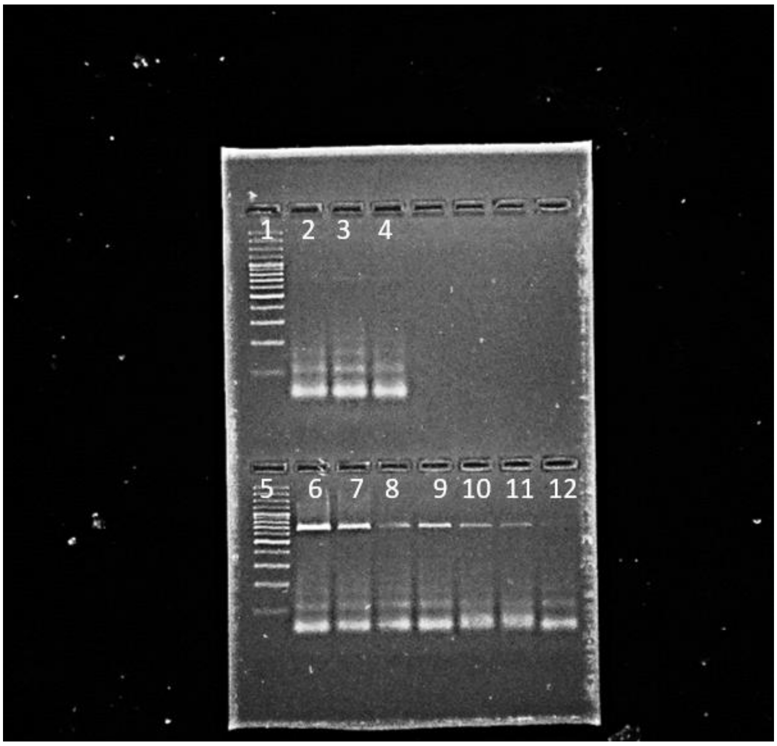

Lane 1: 100 bp plus DNA ladder; 2, 3, and 4: amplicon of DNA extracted through NCM; 5: 100 bp plus DNA ladder, 6, 7, and 8: amplicon of DNA extracted through kit; 9, 10, and 11: amplicon of DNA extracted through CTAB, 12: water control

Gel images of PCR amplification one week after DNA extraction in aphids

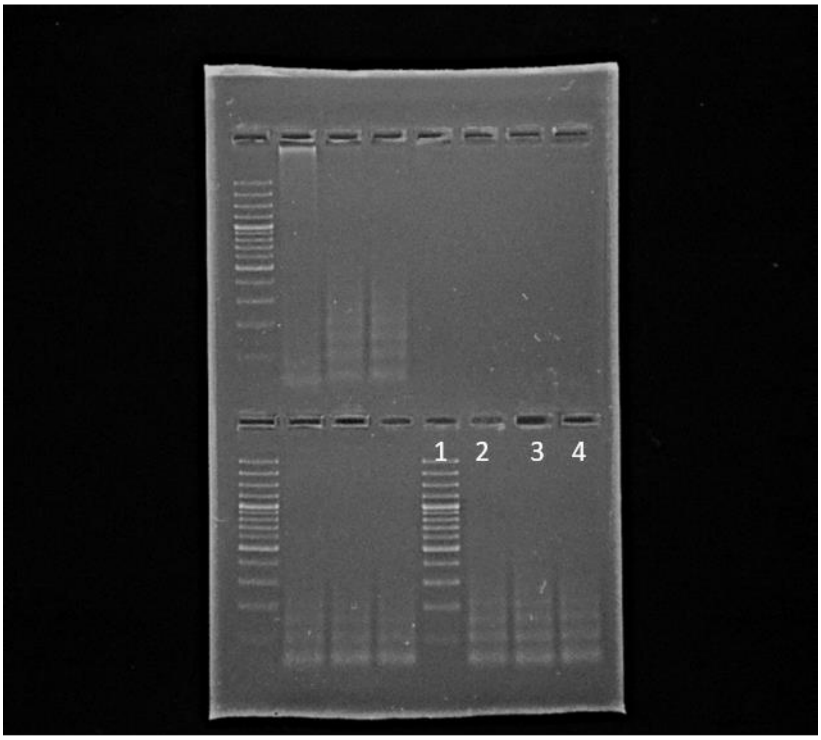

Lane 1: 100 bp plus DNA ladder; 2, 3, and 4: amplicon of DNA extracted through SDW

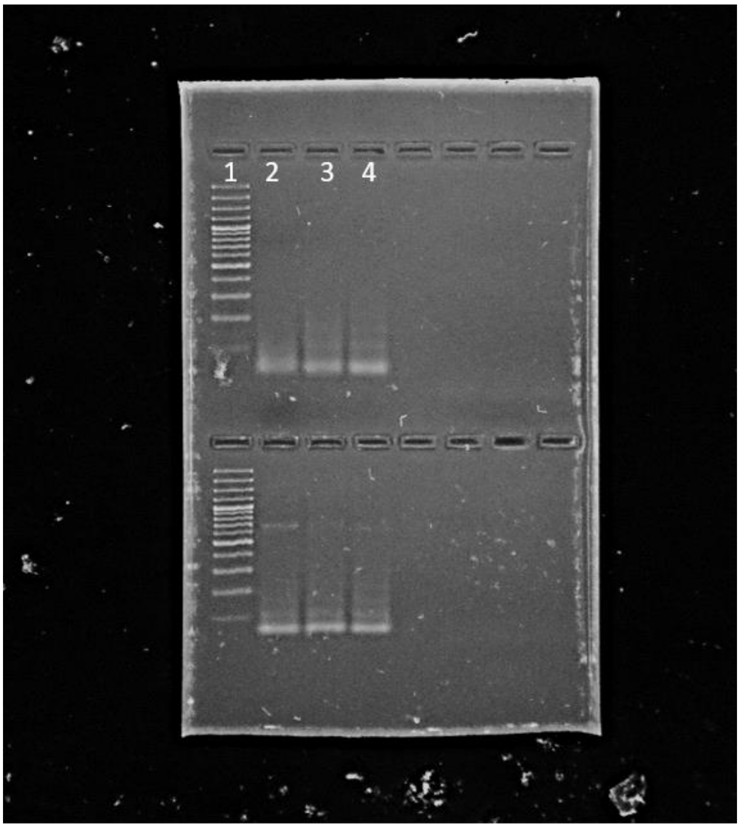

Lane 1: 100 bp plus DNA ladder; 2, 3, and 4: amplicon of DNA extracted through NCM

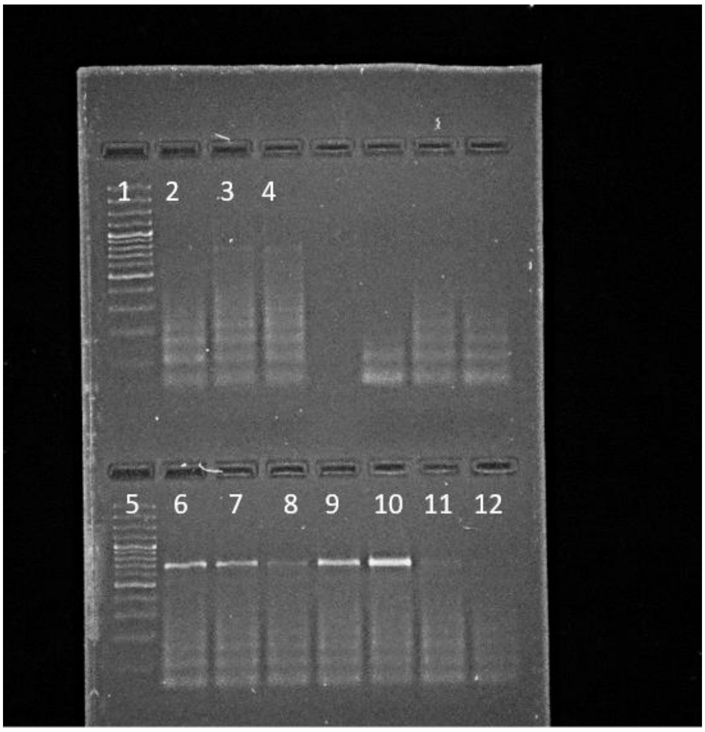

Lane 1: 100 bp plus DNA ladder; 2, 3, and 4: amplicon of DNA extracted through PBS; 5: 100 bp plus DNA ladder, 6, 7, and 8: amplicon of DNA extracted through kit; 9, 10, and 11: amplicon of DNA extracted through CTAB, 12: water control

Gel images of PCR amplification two week after DNA extraction in aphids

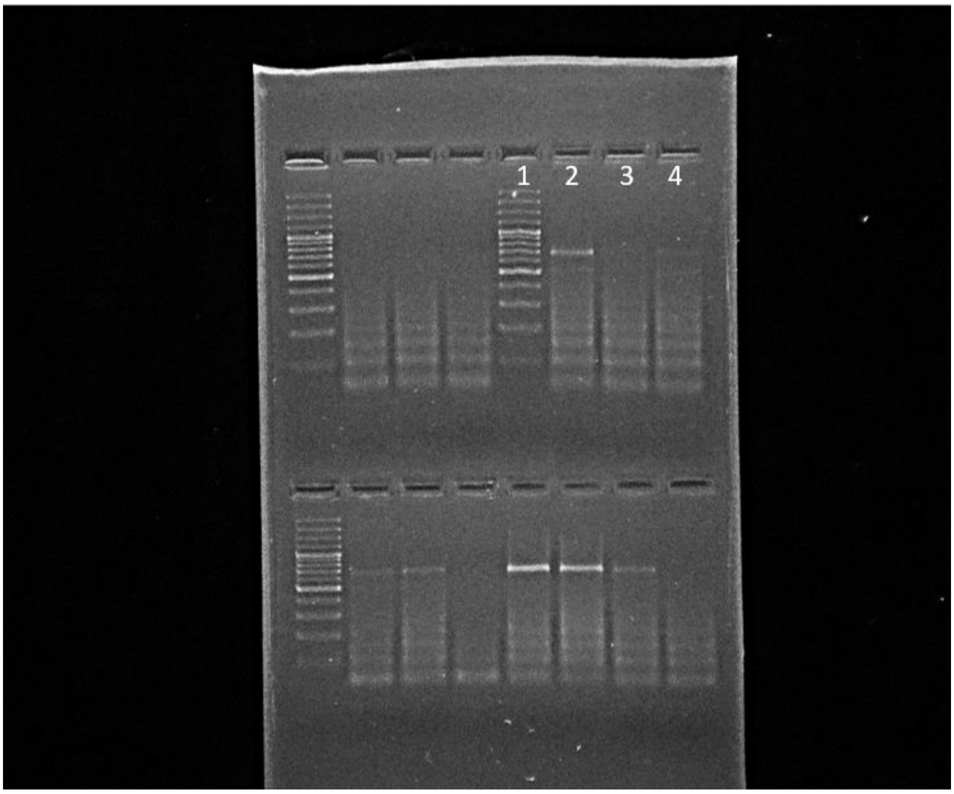

Lane 1: 100 bp plus DNA ladder; 2, 3, and 4: amplicon of DNA extracted through PBS

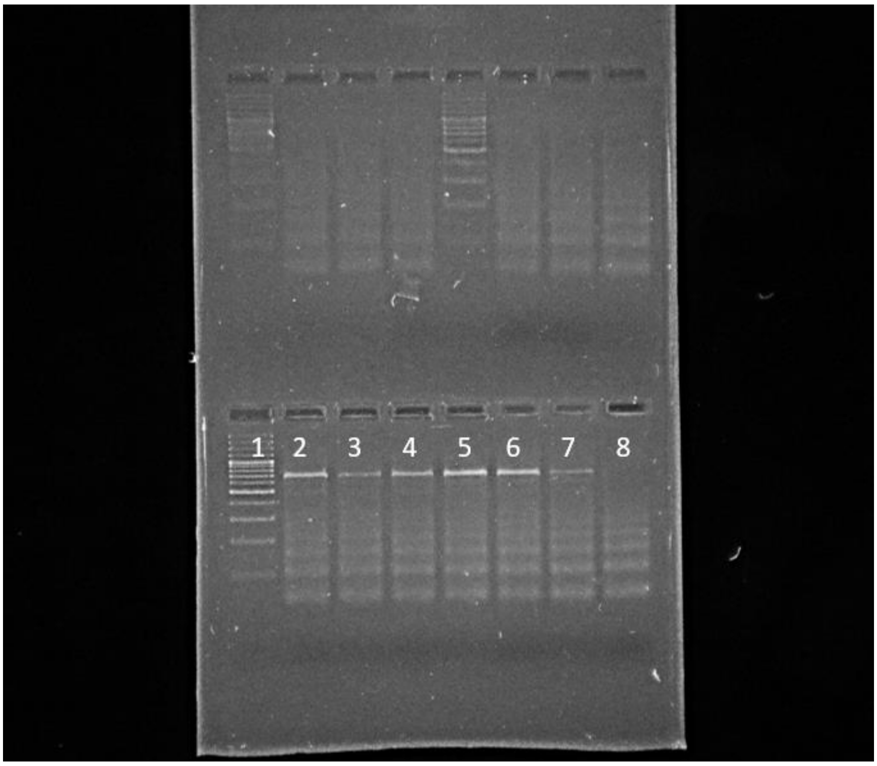

Lane 1: 100 bp plus DNA ladder; 2, 3, and 4: amplicon of DNA extracted through kit; 5, 6, and 7: amplicon of DNA extracted through CTAB; 8: water control

Gel images of PCR amplification three-week after DNA extraction from aphids

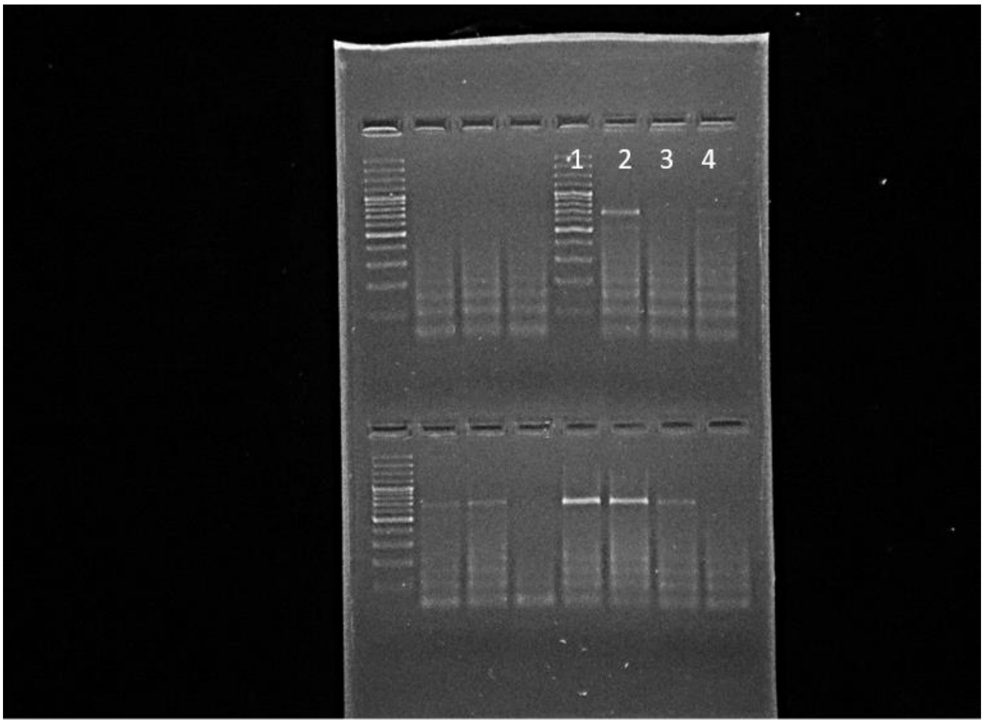

Lane 1: 100 bp plus DNA ladder; 2, 3, and 4: amplicon of DNA extracted through PBS

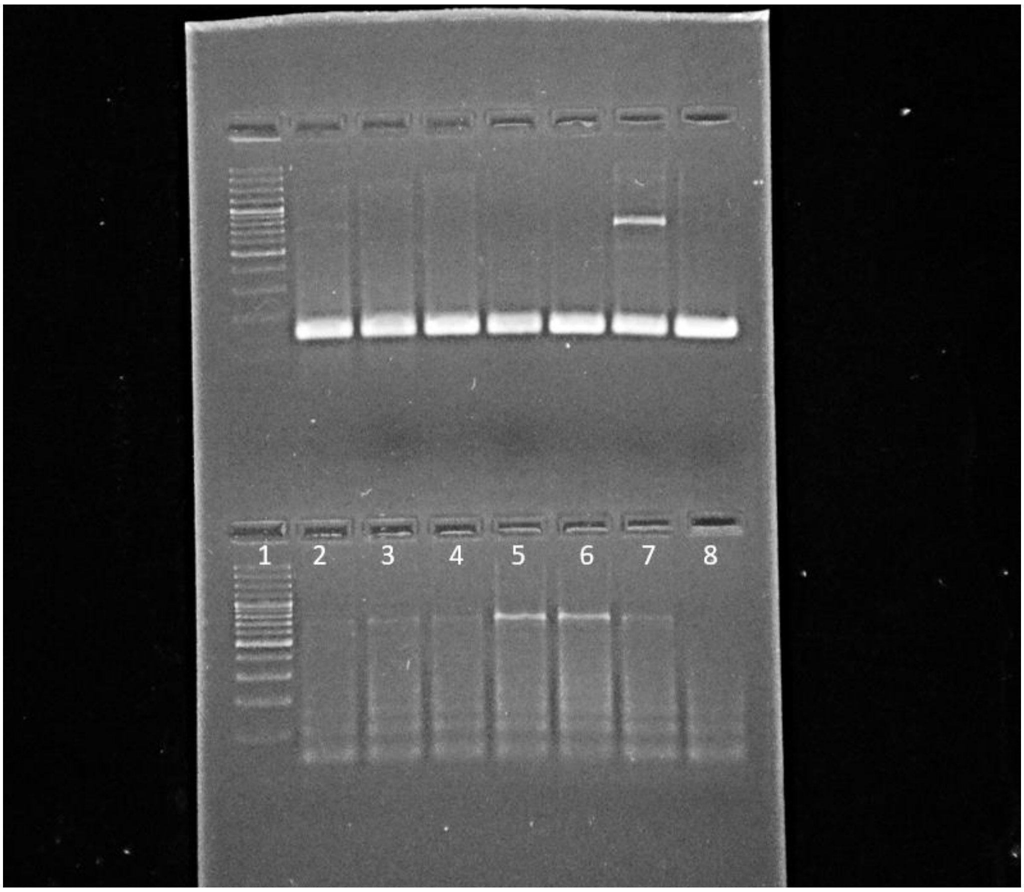

Lane 1: 100 bp plus DNA ladder; 2, 3, and 4: amplicon of DNA extracted through kit; 5, 6, and 7: amplicon of DNA extracted through CTAB; 8: water control

Gel images of PCR amplification four-week after DNA extraction from aphids

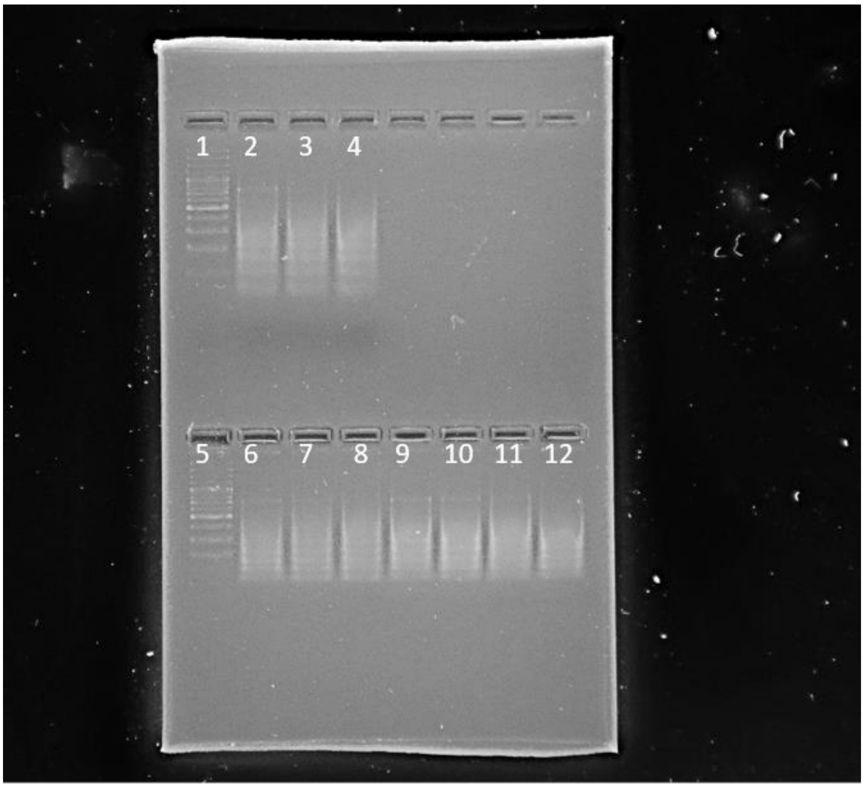

Lane 1: 100 bp plus DNA ladder; 2, 3, and 4: amplicon of DNA extracted through PBS; 5: 100 bp plus DNA ladder, 6, 7, and 8: amplicon of DNA extracted through kit; 9, 10, and 11: amplicon of DNA extracted through CTAB, 12: water control

Thrips

Gel images of PCR amplification immediately after DNA extraction from thrips

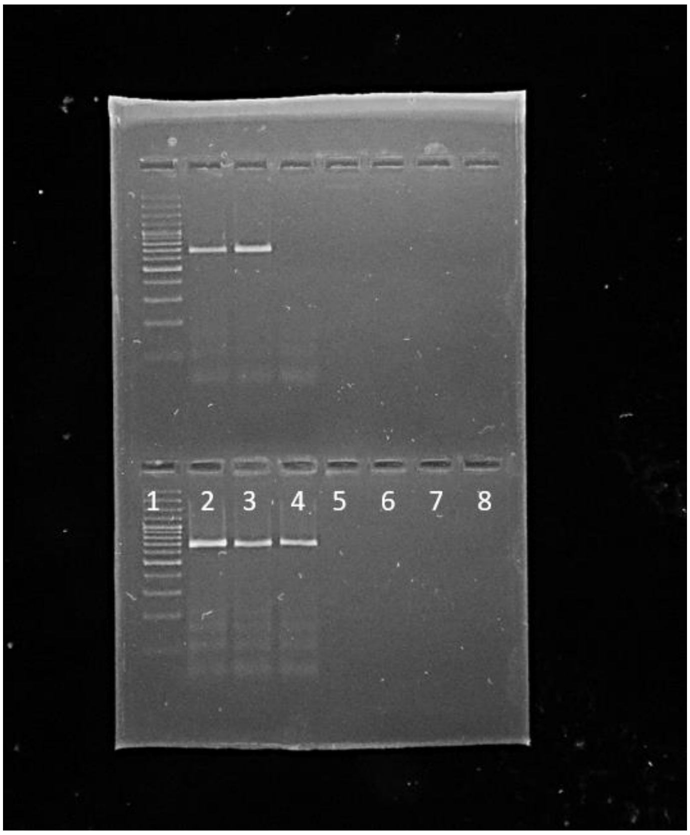

Lane 1: 100 bp plus DNA ladder; 2, 3, and 4: amplicon of DNA extracted through SDW; 5, 6, and 7: amplicon of DNA extracted through NaCl

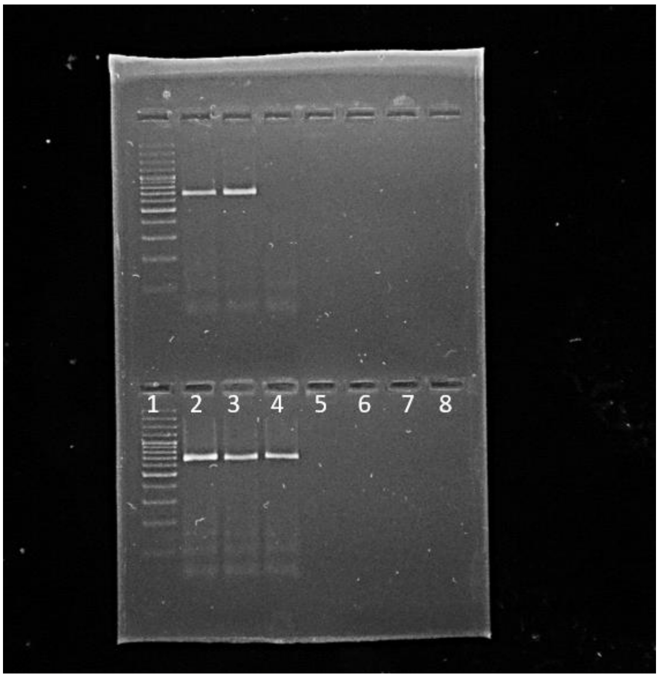

Lane 1: 100 bp plus DNA ladder; 2, 3, and 4: amplicon of DNA extracted through PBS; 5, 6, and 7: amplicon of DNA extracted through EDTA

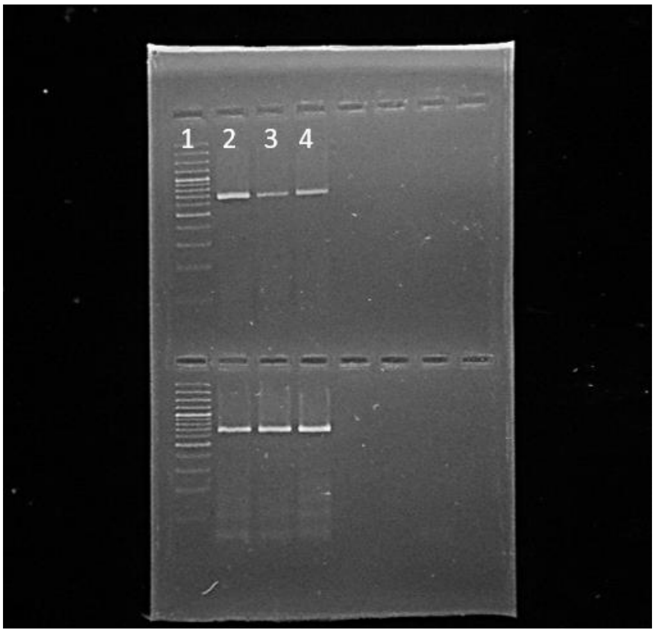

Lane 1: 100 bp plus DNA ladder; 2, 3, and 4: amplicon of DNA extracted through NCM

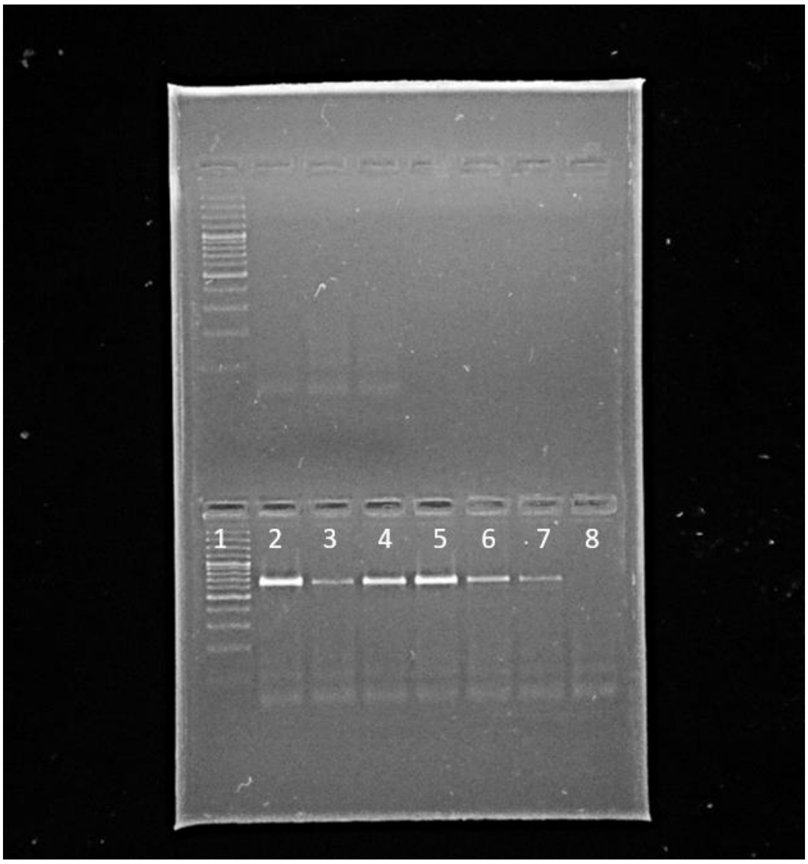

Lane 1: 100 bp plus DNA ladder; 2, 3, and 4: amplicon of DNA extracted through kit; 5, 6, and 7: amplicon of DNA extracted through CTAB; 8: water control

Gel images of PCR amplification one week after DNA extraction from thrips

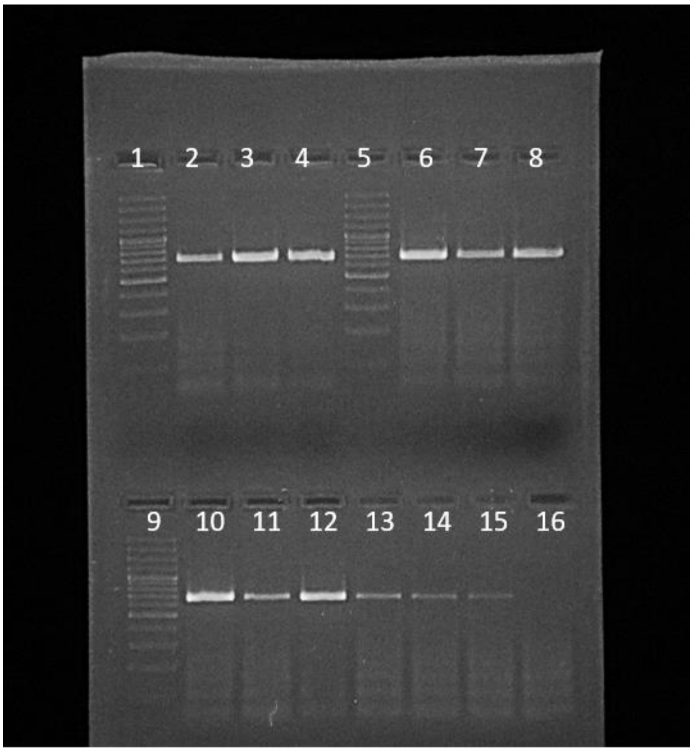

Lane 1: 100 bp plus DNA ladder; 2, 3, and 4: amplicon of DNA extracted through SDW; 5: 100 bp plus DNA ladder; 6, 7, and 8: amplicon of DNA extracted through PBS

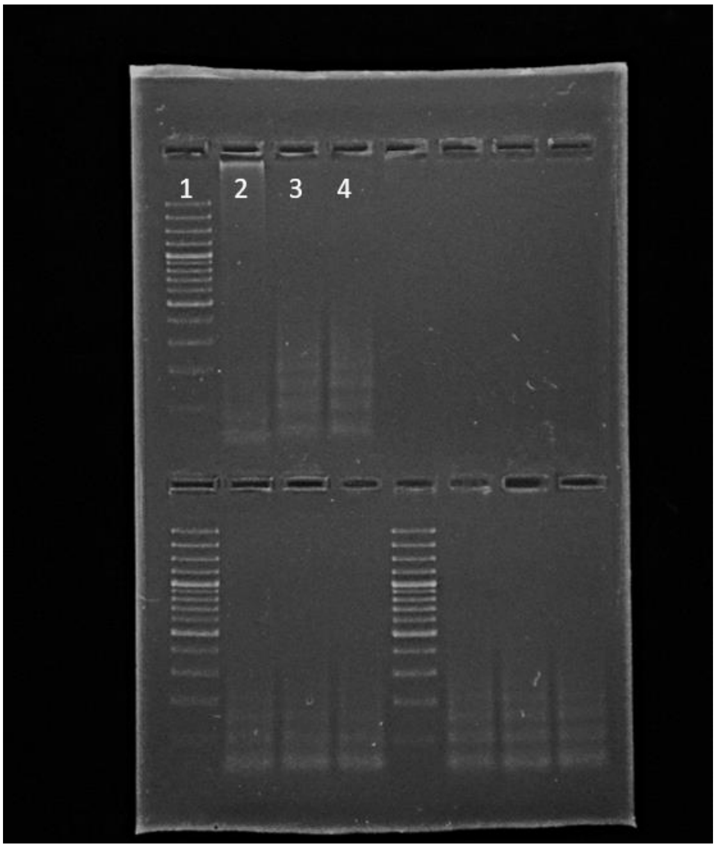

Lane 1: 100 bp plus DNA ladder; 2, 3, and 4: amplicon of DNA extracted through NCM

Gel images of PCR amplification two week after DNA extraction from thrips

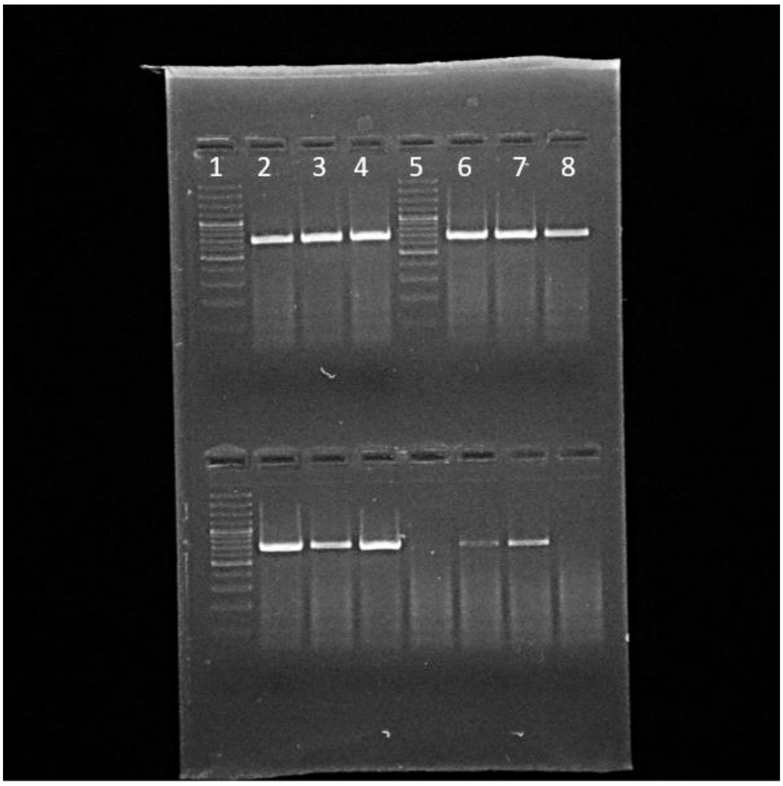

Lane 1: 100 bp plus DNA ladder; 2, 3, and 4: amplicon of DNA extracted through SDW; 5: 100 bp plus DNA ladder; 6, 7, and 8: amplicon of DNA extracted through PBS

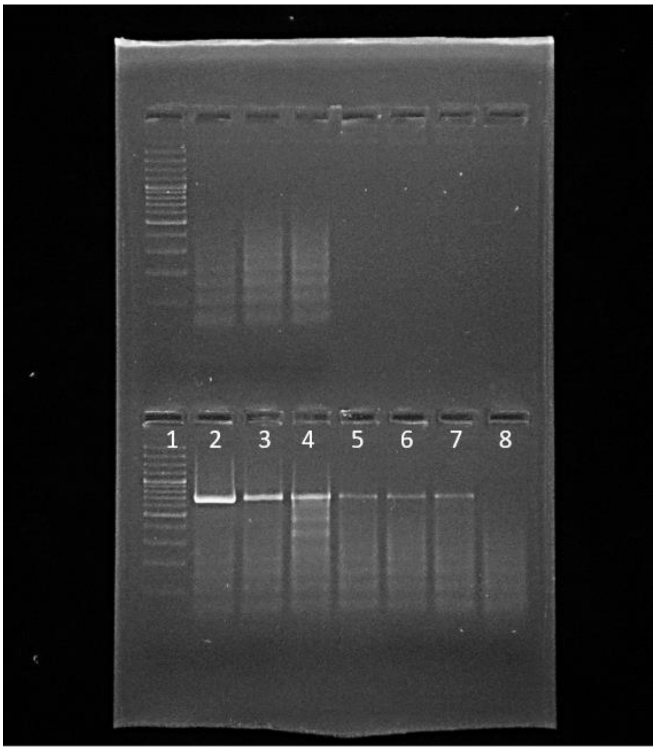

Lane 1: 100 bp plus DNA ladder; 2, 3, and 4: amplicon of DNA extracted through kit; 5, 6, and 7: amplicon of DNA extracted through CTAB; 8: water control

Gel images of PCR amplification three week after DNA extraction from thrips

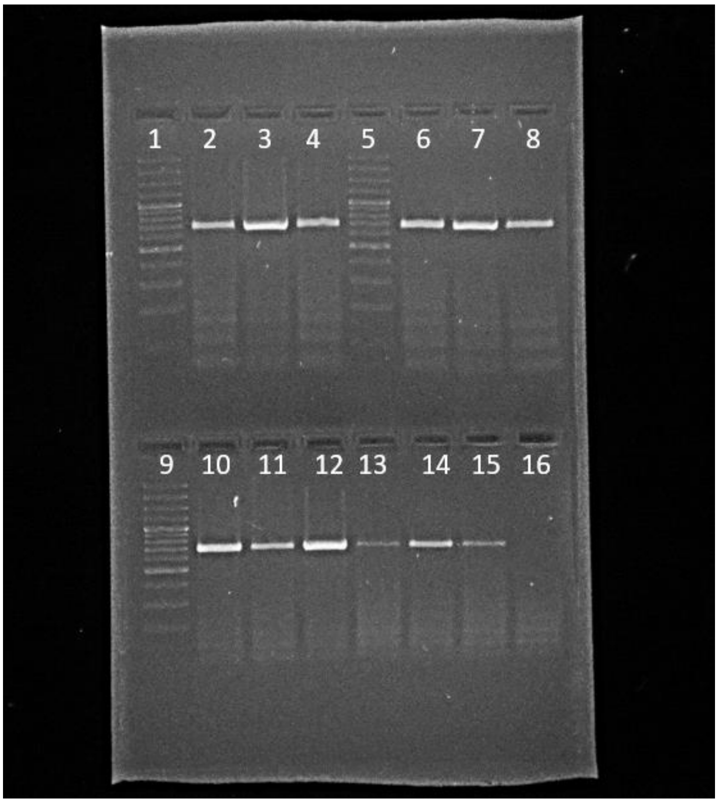

Lane 1: 100 bp plus DNA ladder; 2, 3, and 4: amplicon of DNA extracted through SDW; 5: 100 bp plus DNA ladder; 6, 7, and 8: amplicon of DNA extracted through PBS; 9: 100 bp plus DNA ladder; 10, 11, and 12: amplicon of DNA extracted through kit; 13, 14, and 15: amplicon of DNA extracted through CTAB; 16: water control

Gel images of PCR amplification four week after DNA extraction from thrips

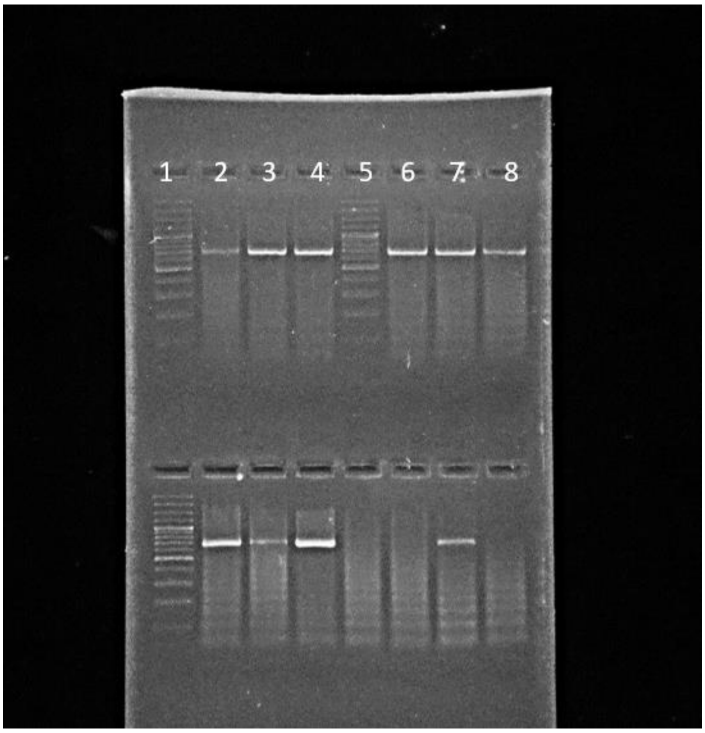

Lane 1: 100 bp plus DNA ladder; 2, 3, and 4: amplicon of DNA extracted through SDW; 5: 100 bp plus DNA ladder; 6, 7, and 8: amplicon of DNA extracted through PBS

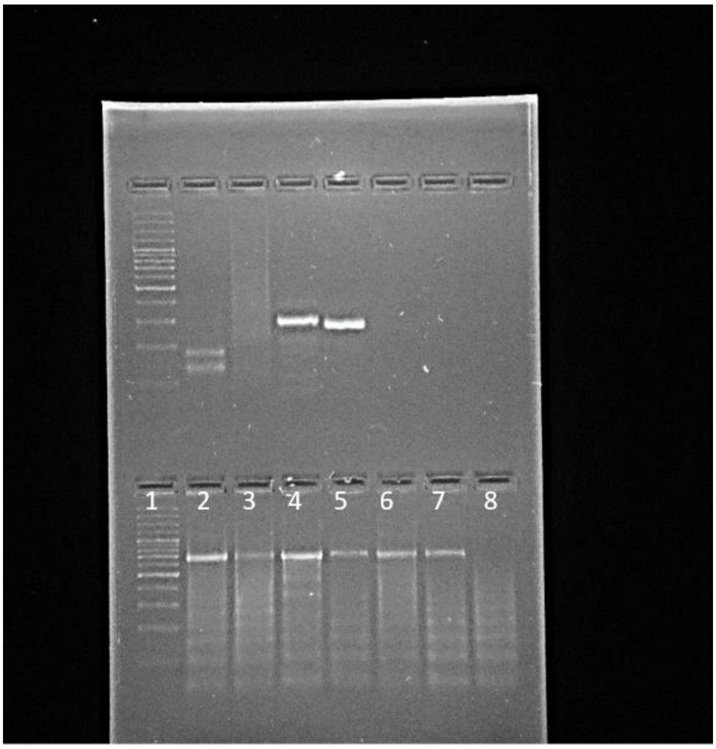

Lane 1: 100 bp plus DNA ladder; 2, 3, and 4: amplicon of DNA extracted through kit; 5, 6, and 7: amplicon of DNA extracted through CTAB; 8: water control

Whitefly

Gel images of PCR amplification immediately after DNA extraction from whitefly

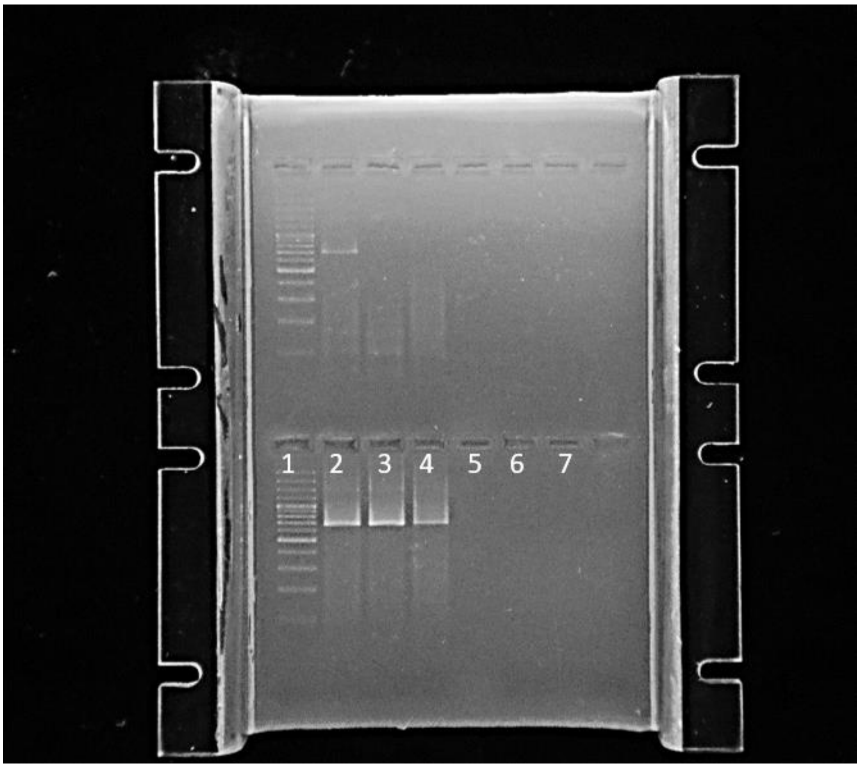

Lane 1: 100 bp plus DNA ladder; 2, 3, and 4: amplicon of DNA extracted through SDW; 5, 6, and 7 amplicon of DNA extracted through NaCl

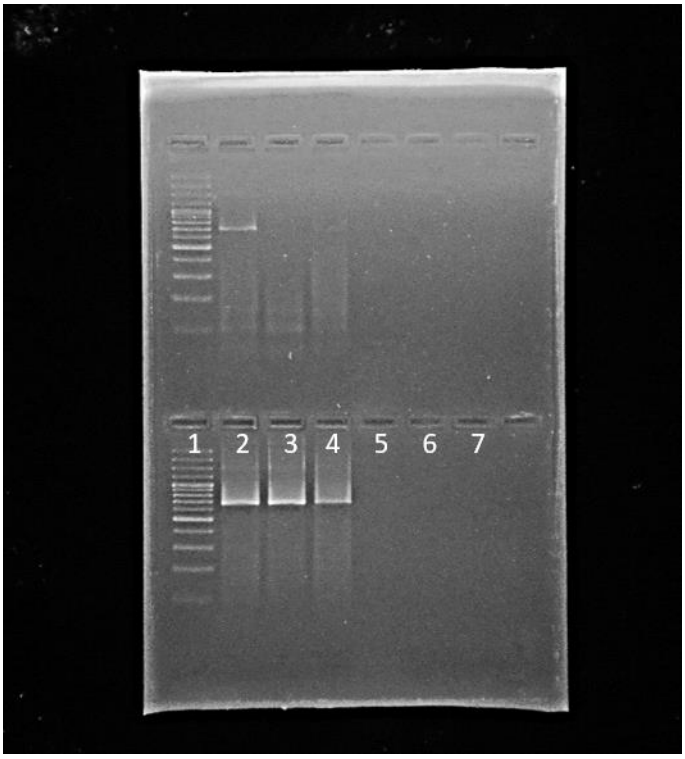

Lane 1: 100 bp plus DNA ladder; 2, 3, and 4: amplicon of DNA extracted through PBS; 5, 6, and 7 amplicon of DNA extracted through EDTA

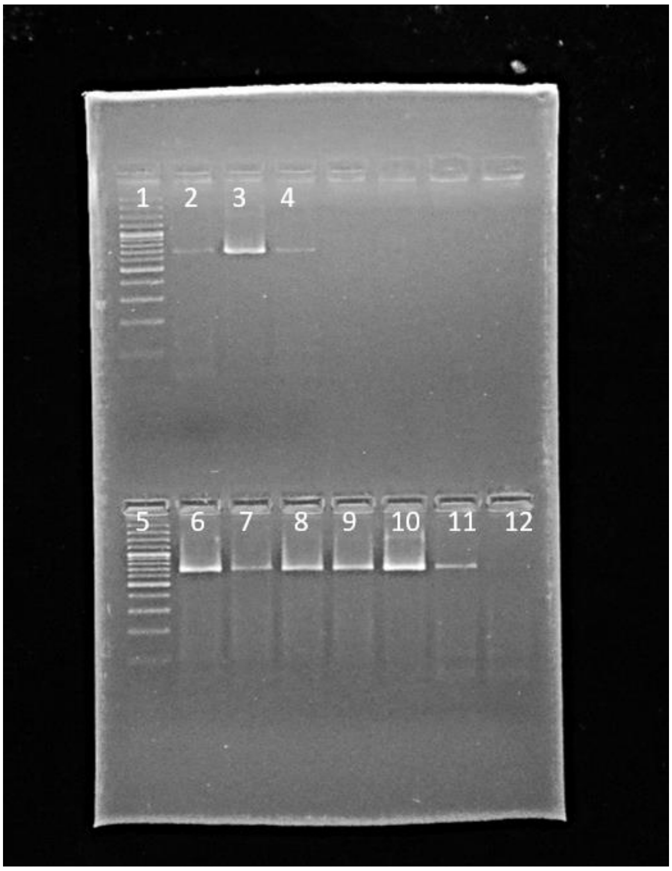

Lane 1: 100 bp plus DNA ladder; 2, 3, and 4: amplicon of DNA extracted through NCM; 5: 100 bp plus DNA ladder; 6, 7, and 8: amplicon of DNA extracted through kit; 9, 10, and 11 amplicon of DNA extracted through CTAB; 12: water control

Gel images of PCR amplification one week after DNA extraction from whitefly

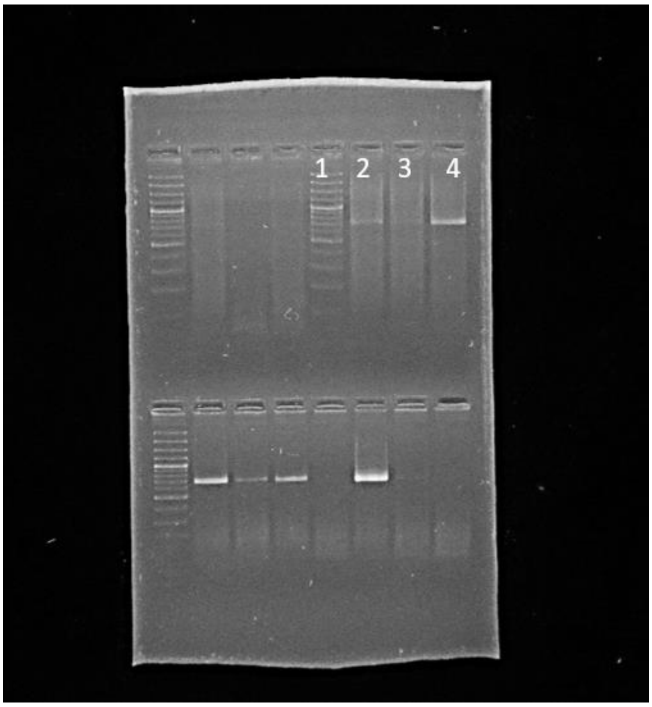

Lane 1: 100 bp plus DNA ladder; 2, 3, and 4: amplicon of DNA extracted through SDW

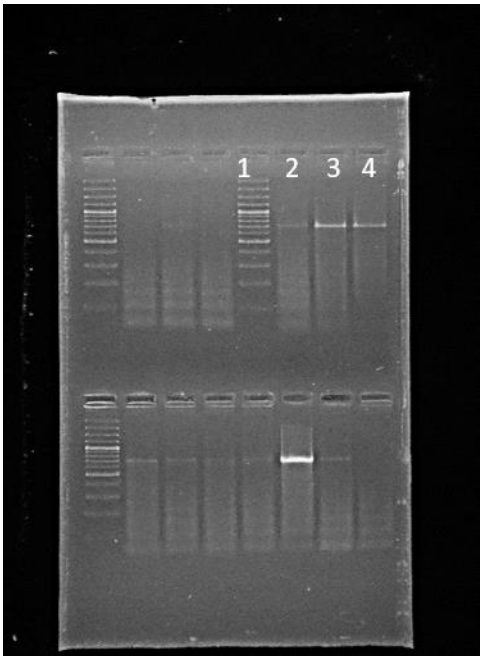

Lane 1: 100 bp plus DNA ladder; 2, 3, and 4: amplicon of DNA extracted through PBS

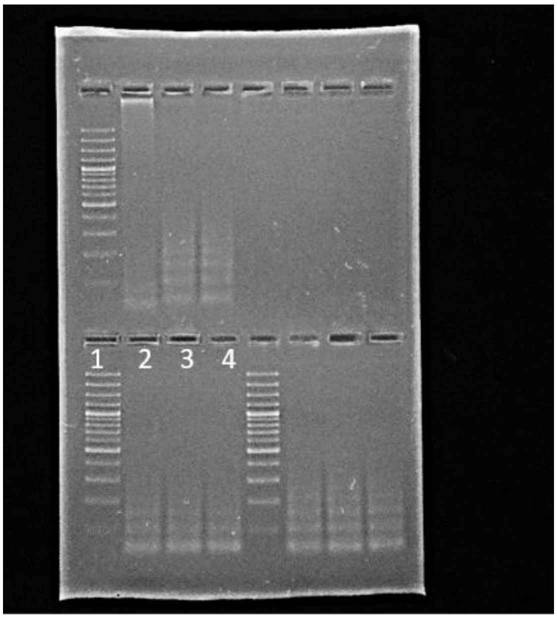

Lane 1: 100 bp plus DNA ladder; 2, 3, and 4: amplicon of DNA extracted through NCM

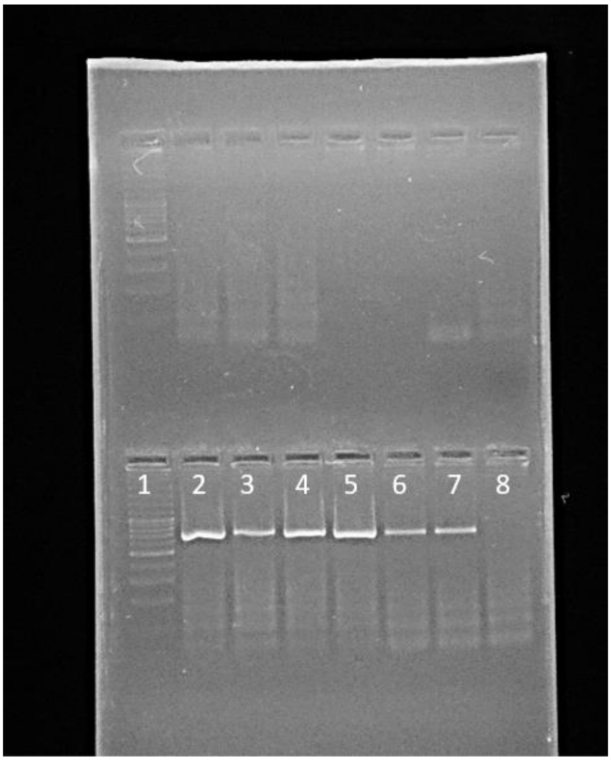

Lane 1: 100 bp plus DNA ladder; 2, 3, and 4: amplicon of DNA extracted through kit; 5, 6, and 7: amplicon of DNA extracted through CTAB; 8: water control

Gel images of PCR amplification two week after DNA extraction from whitefly

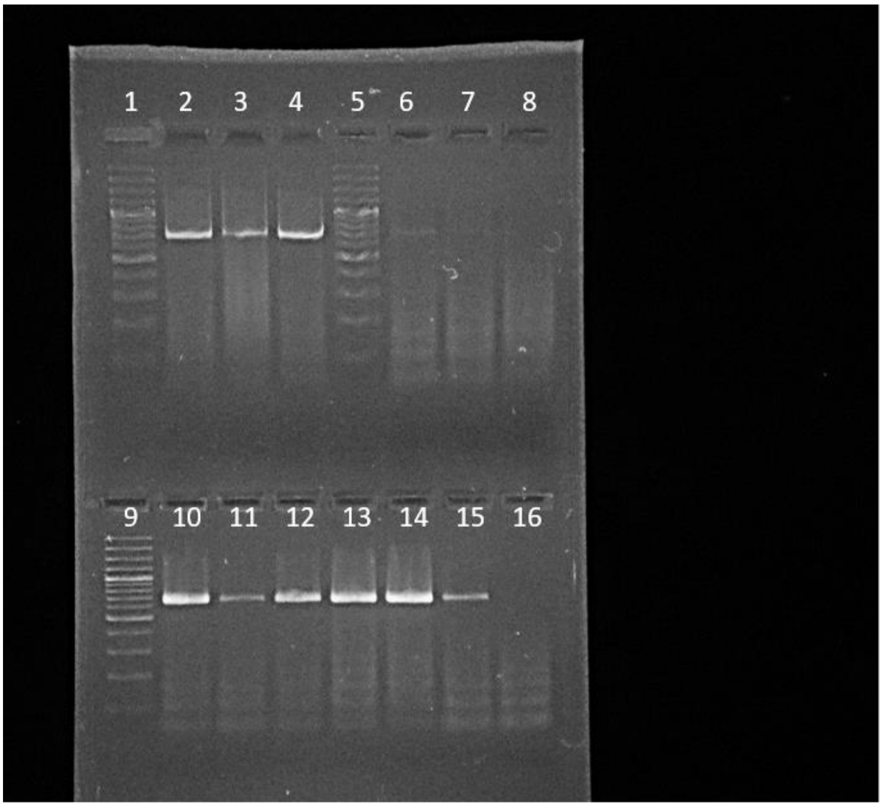

Lane 1: 100 bp plus DNA ladder; 2, 3, and 4: amplicon of DNA extracted through PBS; 5: 100 bp plus DNA ladder; 6, 7, and 8: amplicon of DNA extracted through SDW; 9: 100 bp plus DNA ladder; 10, 11, and 12: amplicon of DNA extracted through kit; 13, 14, and 15: amplicon of DNA extracted through CTAB; 16: water control

Gel images of PCR amplification three week after DNA extraction from whitefly

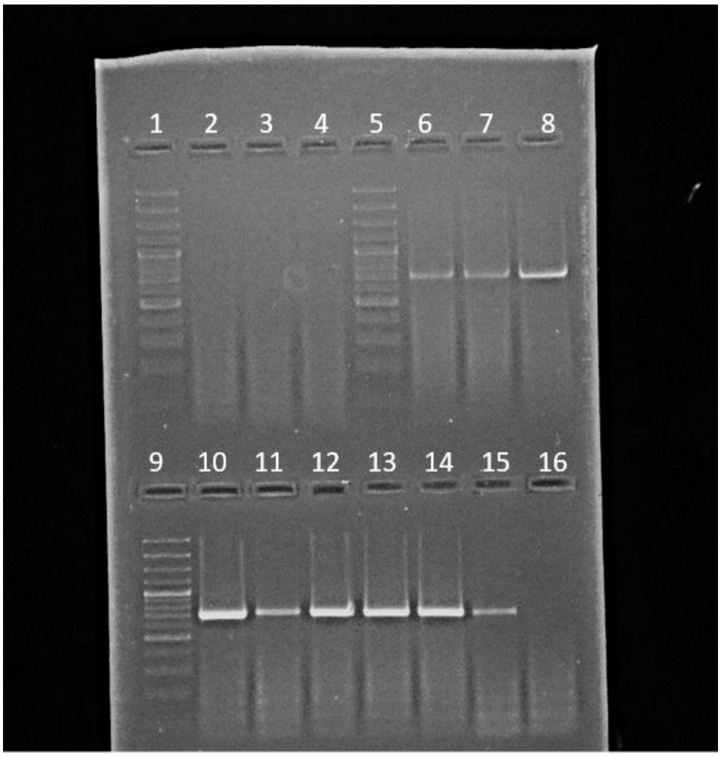

Lane 1: 100 bp plus DNA ladder; 2, 3, and 4: amplicon of DNA extracted through SDW; 5: 100 bp plus DNA ladder; 6, 7, and 8: amplicon of DNA extracted through PBS; 9: 100 bp plus DNA ladder; 10, 11, and 12: amplicon of DNA extracted through kit; 13, 14, and 15: amplicon of DNA extracted through CTAB; 16: water control

Gel images of PCR amplification four week after DNA extraction from whitefly

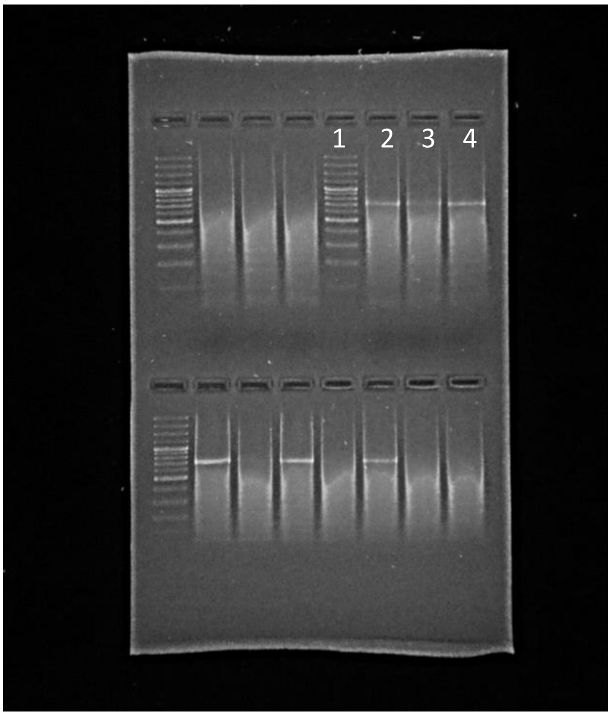

Lane 1: 100 bp plus DNA ladder; 2, 3, and 4: amplicon of DNA extracted through PBS

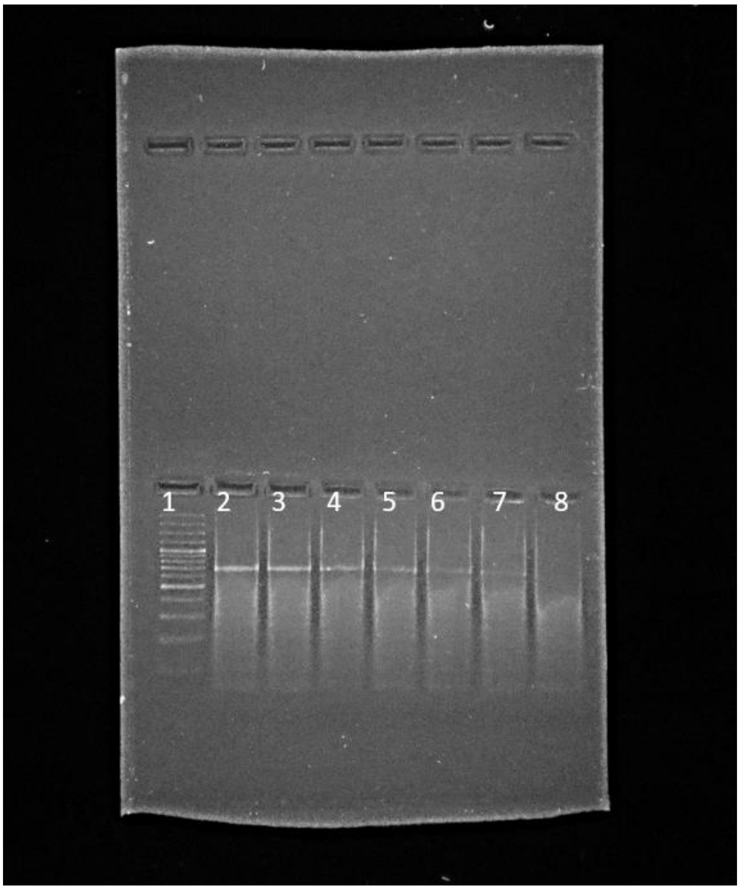

Lane 1: 100 bp plus DNA ladder; 2, 3, and 4: amplicon of DNA extracted through kit; 5, 6, and 7: amplicon of DNA extracted through CTAB; 8: water control

Gel images for pathogen detection

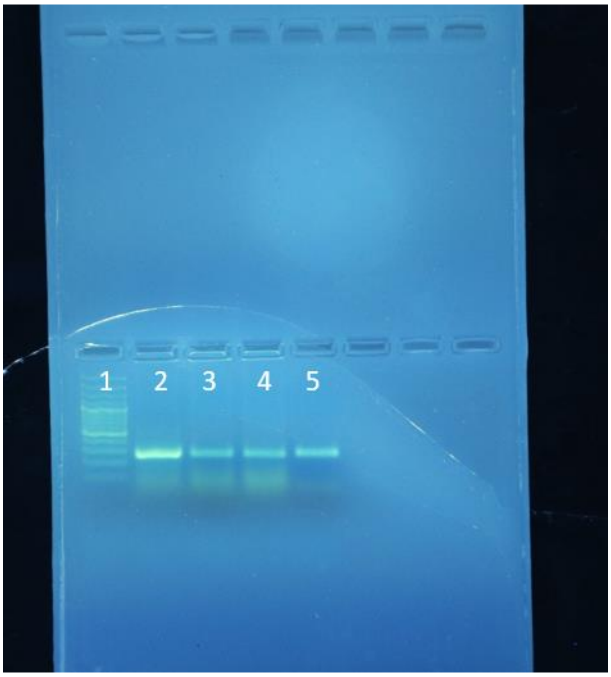

Lane 1: 1 kb plus DNA ladder; 2-5: amplicon of CLcV from whitefly by DNA extracted through SDW method

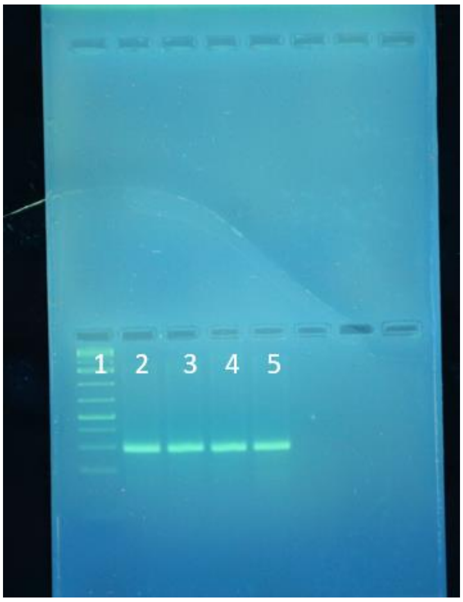

Lane 1: 1 kb plus DNA ladder; 2-5: amplicon of CLcV from whitefly by DNA extracted through PBS method

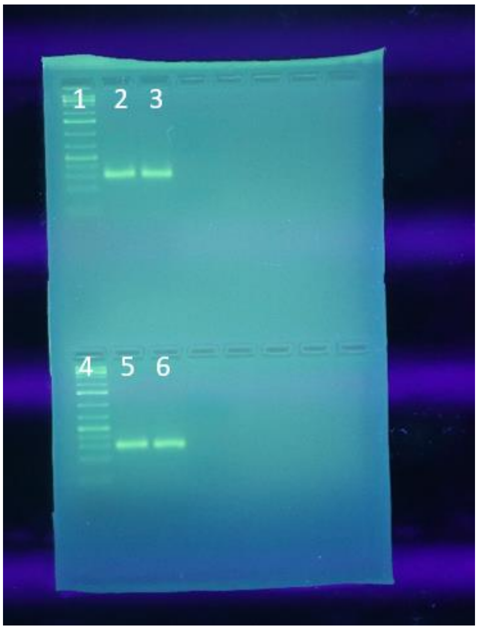

Lane 1: 1 kb plus DNA ladder; 2-3: amplicon of CLcV from whitefly by DNA extracted through kit; 4: 1kb plus DNA ladder; 5-6 amplicon of CLcV from whitefly by DNA extracted through CTAB
