# Supplementary material for: Rapid and zero-cost DNA extraction from soft-bodied insects for routine PCR-based applications
Source: PLoS One. 2022 Jul 15;17(7):e0271312. doi: 10.1371/journal.pone.0271312 (PMC9286237; doi:10.1371/journal.pone.0271312)
Supplement: S3 Fig — (DOCX) [file pone.0271312.s004.docx]

**Whitefly**


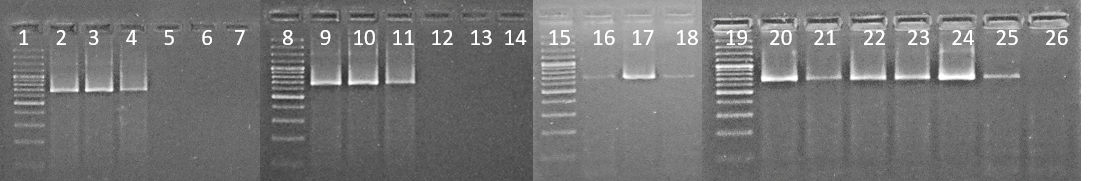


**Supplementary Figure 3a: PCR amplification immediately after DNA extraction**

Lane 1, 8, 15, 19: 100 bp plus DNA ladder; amplicons of DNA extracted through SDW (lane 2-4); NaCl (lane 5-7); PBS (lane 9-11); EDTA (lane 12-14); NCM (lane 16-18); kit (lane 20-22); CTAB (lane 23-25); and water control (lane 26).


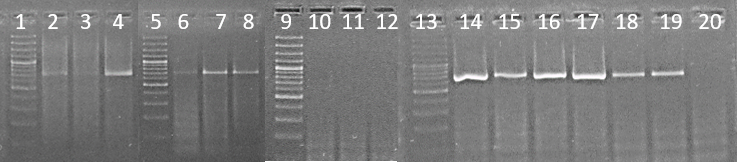


**Supplementary Figure 3b: PCR amplification one-week after DNA extraction**

Lane 1, 5, 9, 13: 100 bp plus DNA ladder; amplicons of DNA extracted through SDW (lane 2-4); PBS (lane 6-8); NCM (lane 10-12); kit (lane 14-16), CTAB (lane 17-19); and water control (lane 20).


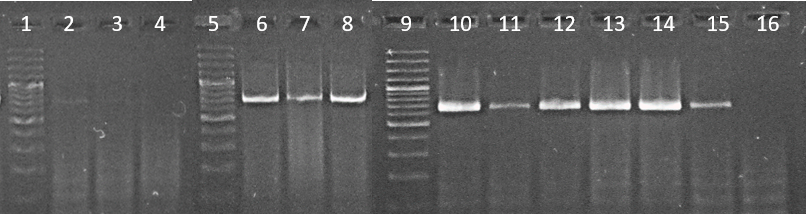


**Supplementary Figure 3c: PCR amplification two-week after DNA extraction**

Lane 1, 5, 9: 100 bp plus DNA ladder; amplicons of DNA extracted through SDW (lane 2-4); PBS (lane 6-8); kit (lane 10-12); CTAB (lane (13-15); and water control (lane 16).


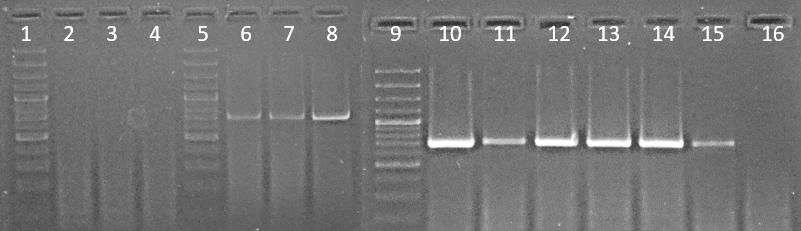


**Supplementary Figure 3d: PCR amplification three-week after DNA extraction**

Lane 1, 5, 9: 100 bp plus DNA ladder; amplicons of DNA extracted through SDW (lane 2-4); PBS (lane 6-8); kit (lane 10-12); CTAB (lane (13-15); and water control (lane 16).


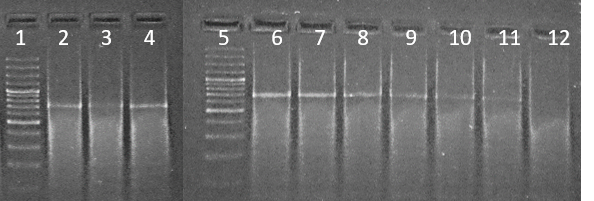


**Supplementary Figure 3e: PCR amplification four-week after DNA extraction**

Lane 1, 5: 100 bp plus DNA ladder; amplicons of DNA extracted through PBS (lane 2-4); kit (lane 6-8); CTAB (lane 9-11); and water control (lane 12).
